# Satellite-based estimates of decline and rebound in China’s CO_2_ emissions during COVID-19 pandemic

**DOI:** 10.1126/sciadv.abd4998

**Published:** 2020-12-02

**Authors:** Bo Zheng, Guannan Geng, Philippe Ciais, Steven J. Davis, Randall V. Martin, Jun Meng, Nana Wu, Frederic Chevallier, Gregoire Broquet, Folkert Boersma, Ronald van der A, Jintai Lin, Dabo Guan, Yu Lei, Kebin He, Qiang Zhang

**Affiliations:** 1 Laboratoire des Sciences du Climat et de l’Environnement, LSCE/IPSL, CEA-CNRS-UVSQ, Université Paris-Saclay, Gif-sur-Yvette, France.; 2 Ministry of Education Key Laboratory for Earth System Modeling, Department of Earth System Science, Tsinghua University, Beijing, China.; 3 State Key Joint Laboratory of Environment Simulation and Pollution Control, School of Environment, Tsinghua University, Beijing, China.; 4 Department of Earth System Science, University of California, Irvine, Irvine, CA, USA.; 5 Department of Civil and Environmental Engineering, University of California at Irvine, Irvine, CA, USA.; 6 Department of Energy, Environmental and Chemical Engineering, Washington University in St. Louis, St. Louis, MO, USA.; 7 Department of Physics and Atmospheric Science, Dalhousie University, Halifax, NS, Canada.; 8 Harvard-Smithsonian Center for Astrophysics, Cambridge, MA, USA.; 9 Royal Netherlands Meteorological Institute (KNMI), De Bilt, Netherlands.; 10 Environmental Sciences Group, Wageningen University, Wageningen, Netherlands.; 11 Nanjing University of Information Science and Technology (NUIST), No. 219, Ningliu Road, Nanjing, Jiangsu, China.; 12 Laboratory for Climate and Ocean-Atmosphere Studies, Department of Atmospheric and Oceanic Sciences, School of Physics, Peking University, Beijing, China.; 13 Chinese Academy of Environmental Planning, Beijing, China.

## Abstract

Changes in CO_2_ emissions during the COVID-19 pandemic have been estimated from indicators on activities like transportation and electricity generation. Here, we instead use satellite observations together with bottom-up information to track the daily dynamics of CO_2_ emissions during the pandemic. Unlike activity data, our observation-based analysis deploys independent measurement of pollutant concentrations in the atmosphere to correct misrepresentation in the bottom-up data and can provide more detailed insights into spatially explicit changes. Specifically, we use TROPOMI observations of NO_2_ to deduce 10-day moving averages of NO*_x_* and CO_2_ emissions over China, differentiating emissions by sector and province. Between January and April 2020, China’s CO_2_ emissions fell by 11.5% compared to the same period in 2019, but emissions have since rebounded to pre-pandemic levels before the coronavirus outbreak at the beginning of January 2020 owing to the fast economic recovery in provinces where industrial activity is concentrated.

## Teaser

Satellite reveals 10-day mean and spatially explicit variations in China’s CO_2_ emissions during and after COVID-19 lockdown.

## INTRODUCTION

In the first half of 2020, most countries in the world have imposed stringent policies to slow down the spread of coronavirus disease 2019 (COVID-19), closing businesses and factories, restricting travel, and issuing stay-at-home orders. Such lockdown measures have helped to curb the spread of the virus (*1*) and, meanwhile, caused large reductions in global demand for fossil fuels (*2*). In turn, levels of nitrogen dioxide (NO_2_) and other air pollutants have also fallen across the globe (*3*–*5*), and global carbon dioxide (CO_2_) emissions declined by an estimated 8.6%, based on indicators of energy use between January and April of 2020 as compared to the same period in 2019 (*6*). If history is a guide, such reductions in air pollution and CO_2_ emissions could be temporary: Global CO_2_ emissions have rebounded and resumed their former rates of growth after every financial crisis in the fossil fuel era (*7*, *8*). Economic activities are already increasing in many places, and governments and central banks have already passed or proposed large economic stimulus packages to spur recovery (*9*) without much consideration for the climate mitigation co-objective so far, especially in the major economies and CO_2_ emitters like China and the United States. Yet, the COVID-19 pandemic has come at an important moment in the centuries-long timeline of fossil energy use, and for that reason as well as the pandemic’s outsized disturbance to social and economic systems (*10*), it may mark a turning point in the world’s energy and economic structure—one with lasting influence on the trajectory of global CO_2_ emissions.

However, annual, country-level inventories of CO_2_ emissions (*11*) are plainly insufficient to monitor variations in the sources and dynamics of CO_2_ emissions during fast-evolving periods like the COVID-19 pandemic. Nor can such annual estimates support adaptive and agile climate and energy policies going forward, as countries and other jurisdictions seek to fine-tune such policies to achieve environmental goals in a rapidly evolving technological and economic context. Increasing the frequency and resolution of data on CO_2_ emissions is thus a research priority. Unfortunately, the data streams required to conduct continuous carbon monitoring with the high temporal and spatial resolution are yet very limited. Bottom-up approaches to estimate daily CO_2_ emissions at the country and sectoral level using activity data and energy use indicators have recently emerged (*6*, *12*, *13*), but gaining access to reliable daily statistics of sector-specific fossil fuel consumption is a challenge, and activity proxies such as traffic congestion indices and heating degree days (HDDs) must be used in those recent studies to empirically analyze the relative changes of emissions. Moreover, such bottom-up methods should match independent atmospheric observations to prove reliability and reduce uncertainties in the daily emission estimates.

Near real-time observations from ground- and space-based platforms thus represent an attractive means of supplementing and validating bottom-up estimates with direct measurements (*14*–*18*). However, CO_2_ concentrations are sparsely sampled in time and space, even for the carbon satellites (*14*, *15*), and natural variability in ecosystem carbon fluxes and atmospheric transport prevents unambiguous detection of fossil fuel CO_2_ emissions over time scales of weeks to months. Moreover, the current generation of carbon monitoring satellites often returns substantial errors in single soundings data (*14*, *15*). Satellite observations of NO_2_, a species co-emitted with CO_2_ during the combustion of fuels, have broader coverage than CO_2_ observations, especially from the recently launched TROPOspheric Monitoring Instrument (TROPOMI) onboard the Copernicus Sentinel-5 Precursor satellite (*19*). Because of the relatively short lifetime in NO*_x_*, the satellite is capable of detecting the short-term variability in NO_2_ columns (*3*, *5*). Satellite NO_2_ columns have been widely used to retrieve the spatial patterns or temporal trends of NO*_x_* emissions [e.g., (*20*–*23*)]. There are also attempts to infer CO_2_ emissions from satellite-based NO_2_ observations (*24*–*26*); however, such a method requires good knowledge of CO_2_-to-NO*_x_* emission ratios that are region dependent, sector specific, and dynamically changing (*27*). The effects of meteorological conditions on observed NO_2_ changes should also be quantified and separated before inferring emissions from observations.

Here, we develop a novel approach to infer a 10-day moving average of anthropogenic CO_2_ emissions from TROPOMI NO_2_ merged with bottom-up information. Details of our data sources and methods are provided in Methods. In summary, we first develop a preliminary bottom-up estimate of sectoral NO*_x_* and CO_2_ emissions in 2020 based on the Multi-resolution Emission Inventory for China (MEIC) in 2019 (*28*) and near real-time statistics and proxies in 2020. On the basis of the high-quality NO_2_ column observations from TROPOMI, we then separate the meteorological effects and model the local sensitivity of NO_2_ column to surface NO*_x_* emission changes (*29*) with the nested-grid GEOS-Chem chemical transport model (*30*) to assess NO*_x_* emission changes in 2020 compared to those in 2019. The meteorological effects on NO_2_ column changes from 2019 to 2020 are quantified through a GEOS-Chem simulation driven by the meteorological field in 2020 with NO*_x_* emissions fixed in 2019, which is similar to the method in (*31*). Next, we use the top-down estimates to correct the sectoral distribution in the preliminary bottom-up emissions, based on the emission differences revealed by the grid cells dominated by a single source sector. Last, the TROPOMI-constrained NO*_x_* emissions and the spatiotemporal heterogeneity of emission sectoral information are combined with the spatially explicit, sector-specific ratio maps of CO_2_-to-NO*_x_* emissions to infer 10-day moving average CO_2_ emissions from specific sectors. We use this integrated satellite-based emissions monitoring approach to track NO*_x_* and CO_2_ emissions in mainland China over the period from January to April 2020 as the coronavirus lockdown was imposed and relaxed.

## RESULTS

### Daily dynamics of NO*_x_* and CO_2_ emissions

**Fig. 1:**
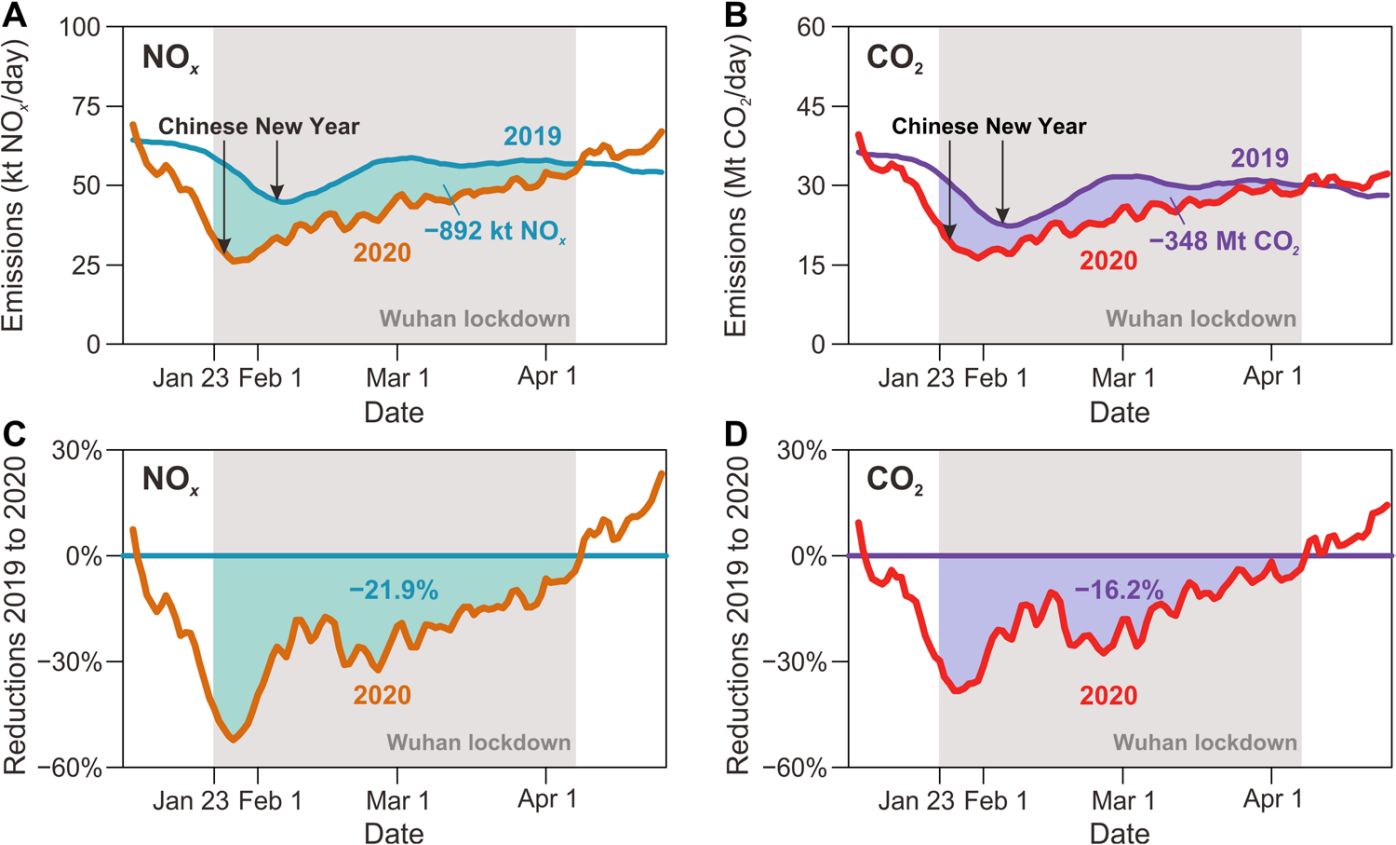
Ten-day moving average NO*_x_* and CO_2_ emissions of China’s national totals from January to April in 2019 and 2020. The 2019 emission data are derived from the MEIC emission inventory, and the 2020 emission data are the TROPOMI-constrained emission estimate from this study. For NO*_x_* (**A** and **C**), the yellow and green curves are for 2020 and 2019, respectively, both of which are plotted according to the date in the *x* axis. The *y* axis in (A) and (C) represent the 10-day moving average of NO*_x_* emissions and the NO*_x_* emission reductions from 2019 to 2020, respectively. (**B**) and (**D**) are plotted for the CO_2_ emissions, which are similar to (A) and (C), respectively. The gray shades reflect the period of the Wuhan lockdown from 23 January 2020 to 7 April 2020.

Figure 1 presents 10-day moving averages of NO*_x_* (Fig. 1, A and C) and CO_2_ emissions (Fig. 1, B and D) of China’s national totals from January to April in 2019 and 2020. The 2019 emissions are directly derived from the MEIC inventory, which is also the base for estimating the NO*_x_* and CO_2_ emissions in 2020 constrained by TROPOMI observations. The 2019 emission data show a reduction in the daily NO*_x_* and CO_2_ by 30 and 38%, respectively, from 1 January to the Chinese New Year 2019 (5 February) and a rebound in emissions 20 days later when the holiday ended and people returned to work. The larger decrease in CO_2_ than in NO*_x_* is because the major driver of emission decrease is the reduced activities in the power and industry sectors (where emission ratios of CO_2_ to NO*_x_* are large), while transport emissions (with low CO_2_-to-NO*_x_* emission ratios) decreased much less because travel demand was still high during the holiday.

The 2020 daily emissions of China constrained by TROPOMI observations, however, show much faster decreases than those in 2019. During the period from 1 January to the Chinese New Year 2020 (25 January), NO*_x_* and CO_2_ emissions are estimated to have dropped by 58 and 51%, respectively. The larger decrease in NO*_x_* emissions than in CO_2_ is due to a much larger decline in the transport emissions than power and industrial emissions during the COVID-19 lockdown as we will show in the following analysis. The daily emissions of NO*_x_* and CO_2_ in 2020 are estimated up to be 50 and 40%, respectively, lower than those in 2019, and they did not return to the preholiday levels until 2 months later.

The divergence of China’s daily emissions between 2020 and 2019 corresponds to the timeline of the virus control measures. The sharper emission declines in 2020 started on 20 January 2020, when the most stringent control measures were activated by the National Health Commission. The Wuhan lockdown began 3 days later on 23 January 2020, which was followed by similar measures in the other Chinese cities within the next few days. These lockdown measures did not ease until about 1 month later when the lowest risk regions and cities slowly reopened some of the less exposed industries and businesses. About 2 months after the Chinese New Year 2020, most of China’s cities had lifted the control measures including Wuhan that reopened on 8 April after a 76-day lockdown. During the Wuhan lockdown period (gray shades in Fig. 1), China’s emissions were lower than the 2019 emissions by a cumulative of 892 kt (thousand metric tons) NO*_x_* (21.9% net reduction; green shades in Fig. 1) and 348 Mt (million metric tons) CO_2_ (16.2% net reduction; blue shades in Fig. 1).

The total reduction in China’s CO_2_ emissions over January to April 2020 is equivalent to a −11.5% decrease over the corresponding period of 2019, a bit higher than the estimate of −7.8% (−3.6 to −12.9%) from (*6*). The largest emission reductions occurred in February, while the emissions rapidly rebounded in March and April (table S1), and the CO_2_ emissions in April 2020 are estimated to rebound to emission levels in 2019. The emission reductions over January to March 2020 are −15.6% compared to the same period of 2019, which is higher than the bottom-up estimate of −10.3% from (*13*). Overall, our TROPOMI-constrained emission estimates present larger emission reductions during January to April than the bottom-up estimates (*6*, *13*).

**Fig. 2:**
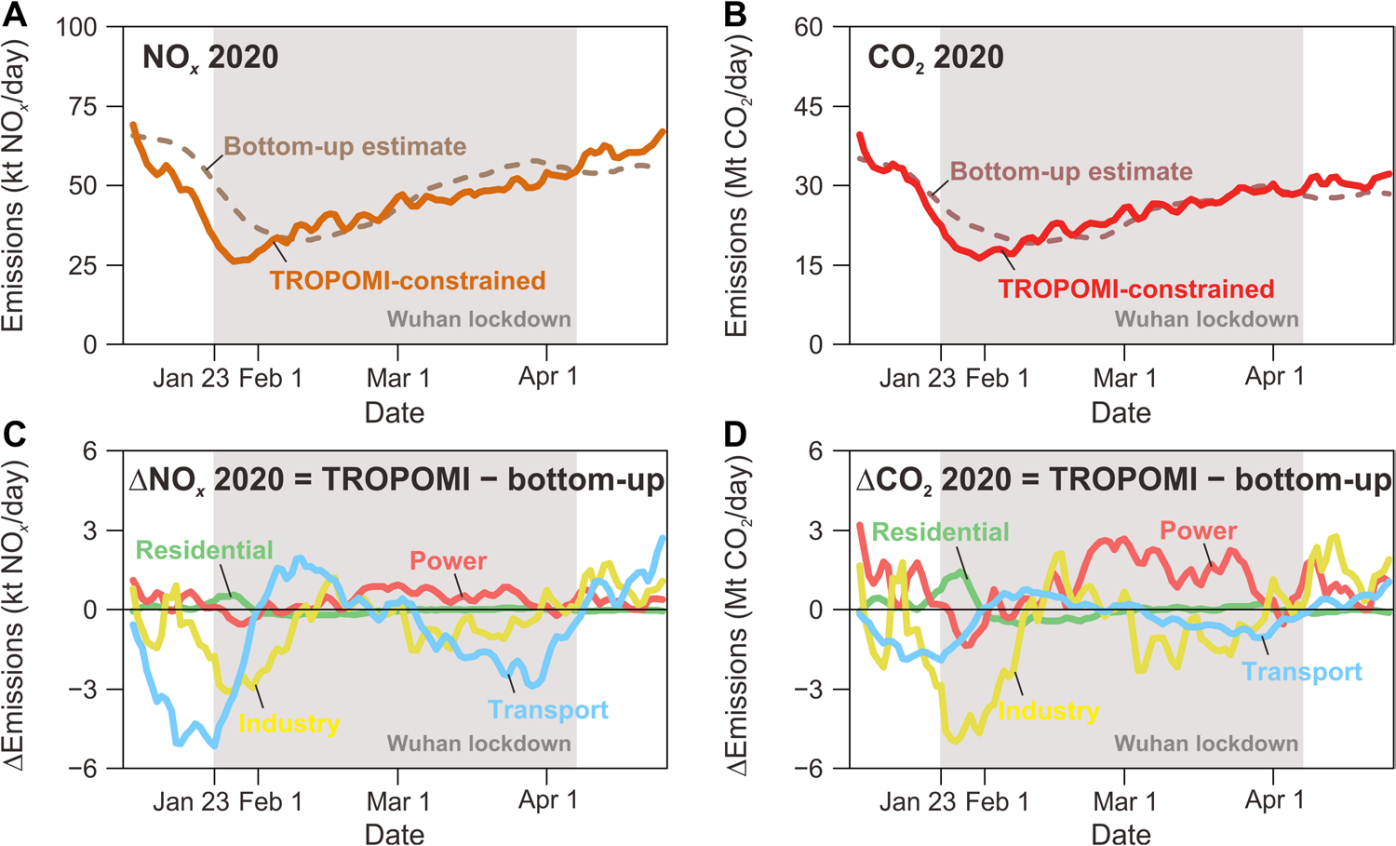
Comparisons between TROPOMI-constrained and bottom-up estimated NO*_x_* and CO_2_ emissions of China’s national totals. The solid curves in (**A**) and (**B**) are the 10-day moving average of TROPOMI-constrained NO*_x_* and CO_2_ emissions, respectively, from January 2020 to April 2020. The dashed curves in (A) and (B) are the bottom-up estimates. The difference of the 10-day moving average emissions between the TROPOMI-based and bottom-up estimates is plotted for NO*_x_* in (**C**) and for CO_2_ in (**D**) for the source sectors of power (red), industry (yellow), residential (green), and transport (blue).

We also used the bottom-up approach (see Methods) to estimate daily NO*_x_* and CO_2_ emissions in China’s national totals in 2020 (dashed curves in Fig. 2, A and B), which present slightly higher emission reductions than (*6*) and (*13*). Our bottom-up results are comparable to the TROPOMI-constrained inversions but still reveal large discrepancies. The differences in the NO*_x_* emissions mainly occur over the regions dominated by the emissions from transport (blue curve in Fig. 2C) and industry (yellow curve in Fig. 2C), while the power sector also contributes to the discrepancy in the CO_2_ emissions (red curve in Fig. 2D). The bottom-up inventory tends to overestimate industrial and transport emissions during the lockdown period, especially at the beginning of lockdown, but tends to underestimate emissions for the power sector when lockdown was gradually lifted in March. The complete statistics of daily fuel combustion are not available for the bottom-up inventory (see Methods), which relies instead on the daily data for 12% of China’s power plants and some proxies such as the daily traffic congestion indices and monthly industrial gross domestic product (GDP) combined with the daily coal use in the power sector to represent the relative changes of daily emissions, which is inevitably associated with uncertainties.

### Drivers of CO_2_ emissions drop and rebound

**Fig. 3:**
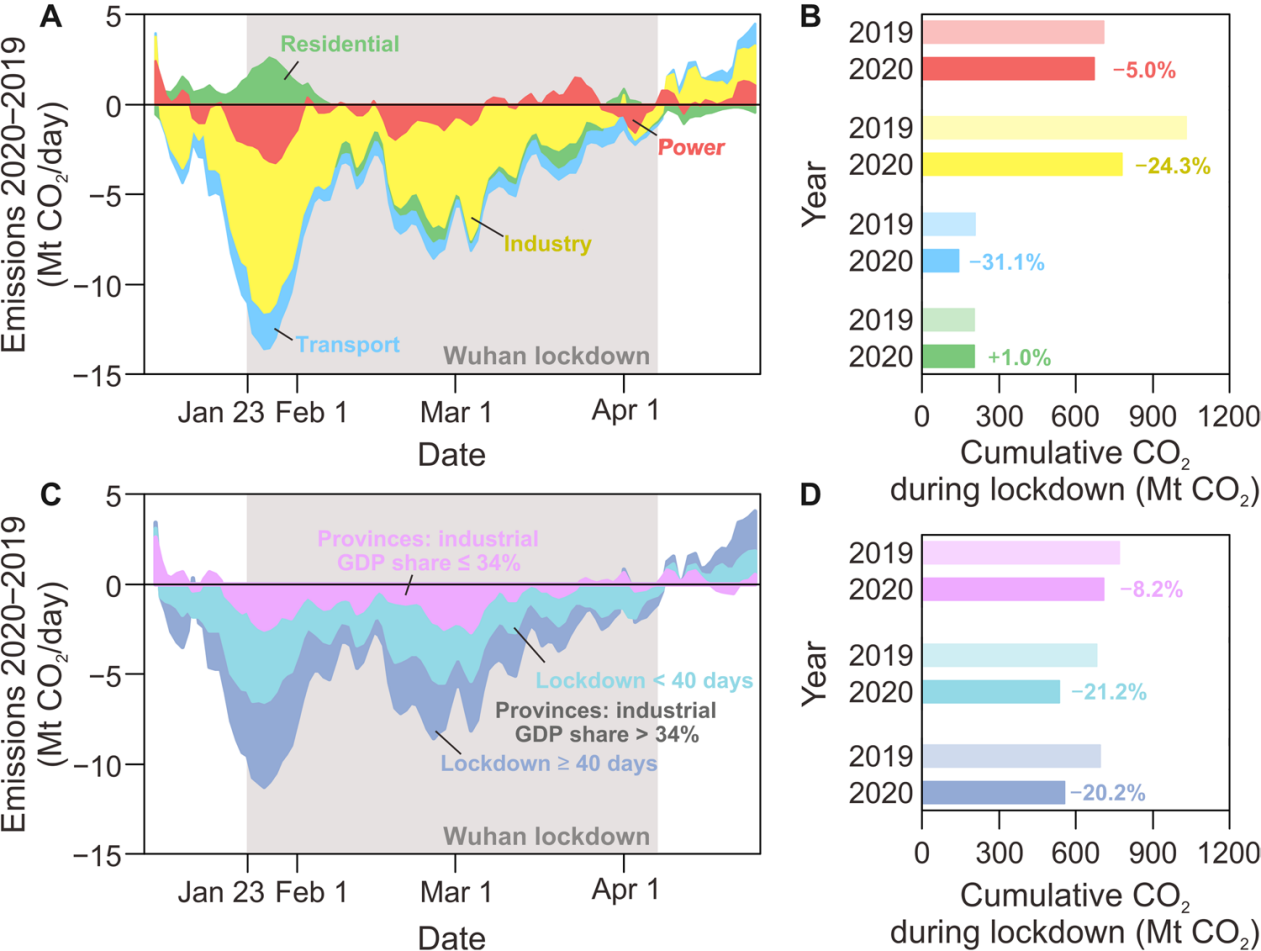
Decomposition of the difference in the 10-day moving average of China’s CO_2_ emissions between 2019 and 2020 by source sector and source region. The emission difference is split into power, industry, residential, and transport sectors in (**A**) and split into three regional categories in (**C**), including (i) the provinces with the share of industrial GDP in provincial total GDP lower than 34%, (ii) the provinces with a share of industrial GDP higher than 34% and a lockdown shorter than 40 days, and (iii) the provinces with the industrial GDP share higher than 34% and a lockdown longer than 40 days. The cumulative CO_2_ emissions during Wuhan lockdown [gray shades in (A) and (C)] are presented by source sector in (**B**) and source region in (**D**).

We decompose the difference in the 10-day moving average of China’s CO_2_ emissions between 2020 and 2019 into power, industry, transport, and residential sectors (Fig. 3, A and B). The industry sector was the major driver of China’s CO_2_ emission changes. During the Wuhan lockdown period (gray shades in Fig. 3), the cumulative CO_2_ emissions from the industry sector declined by 24.3% compared to those in 2019, accounting for 72% of the total reduction in CO_2_ emissions for that period. The transport and power sectors are estimated to have experienced cumulative CO_2_ emission reductions by 31.1 and 5.0%, respectively, contributing 18 and 10% to the total CO_2_ reduction. The emission decline in the power sector was possibly driven by the lower demand by industry that consumes more than two-thirds of the total electricity in China. Residential emissions increased by 1%. The population-weighted HDD in 2020 winter was estimated 3% lower than that in 2019 due to the high air temperature; therefore, the larger residential emissions in 2020 are mainly due to the more energy consumed when people were forced to stay at home, especially at the beginning of the lockdown period. After the lockdown measures were lifted, the recovery of the industry and transport sectors rapidly pushed CO_2_ emissions back to pre-lockdown levels at the beginning of January in 2020.

The regional decomposition (Fig. 3, C and D) also confirms a CO_2_ emission dynamics that was dominated by industry. China’s provinces were classified into three categories distinguished by its increasing share of industrial GDP in the provincial total GDP and the length of lockdown. The provinces dominated by the industrial economy were more sensitive to the lockdown measures, with both a deeper drop and a faster recovery in emissions. The CO_2_ emissions declined by 8.2% in the provinces where industrial GDP contributes less than 34% (median value of all the provinces) of the provincial total GDP (pink bars in Fig. 3, C and D). However, the emission decreases were more than twice higher in provinces where the share of industrial GDP is larger than 34%. The industrial provinces with a lockdown longer than 40 days (dark blue bars in Fig. 3, C and D) show comparable reductions of CO_2_ emissions (−20.2%) than those (−21.2%) with a shorter lockdown period (light blue bars in Fig. 3, C and D).

### Response of provincial emissions to COVID-19

**Fig. 4:**
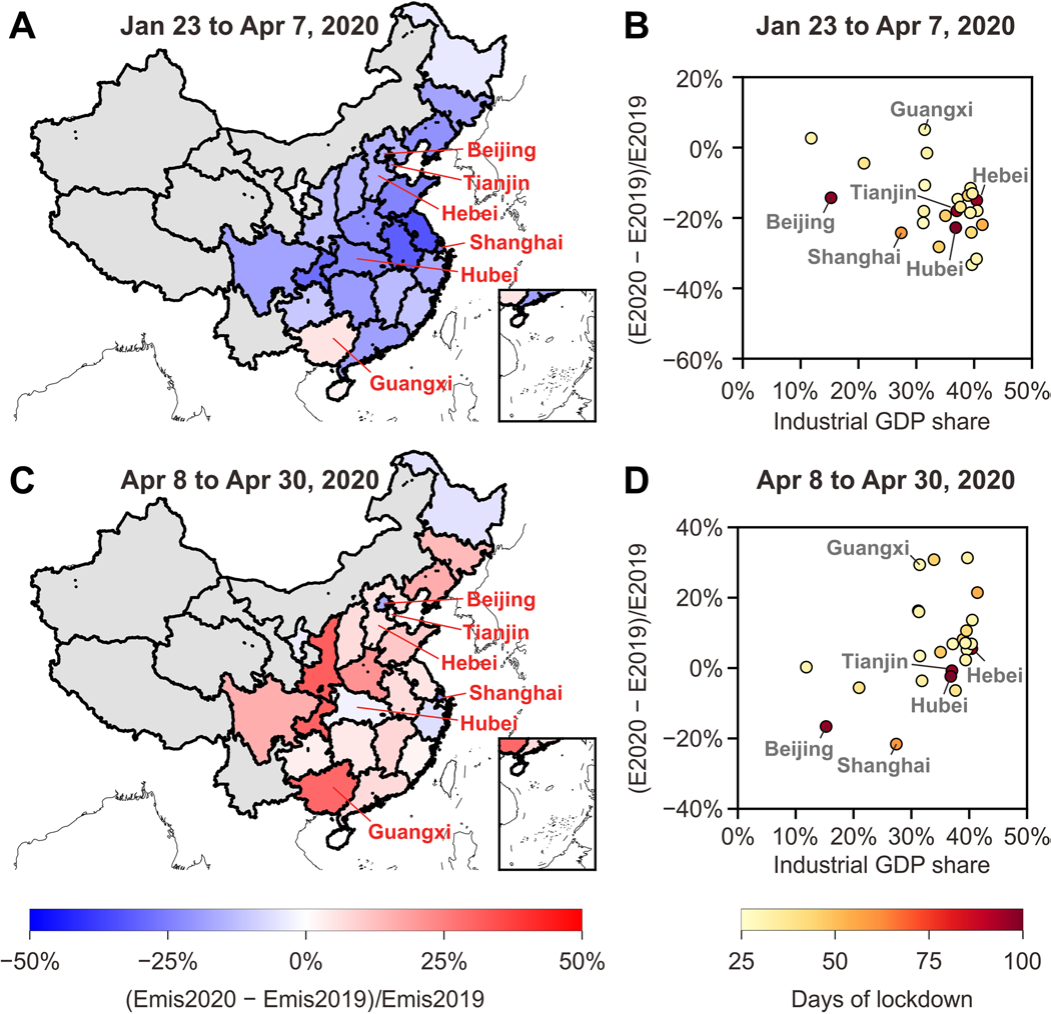
Relative changes in provincial CO_2_ emissions of mainland China from 2019 to 2020. The relative changes in the cumulative CO_2_ emissions between 23 January and 7 April are presented in (**A**) and (**B**). We only plot the provinces with enough TROPOMI observations used in this study (NO_2_ TVCDs larger than 1 × 10^15^ molecules/cm^2^) that covered more than 80% of their anthropogenic NO*_x_* emissions from January to April in 2019 and 2020. The provinces without enough TROPOMI observations are plotted with the gray color. The colors of other provinces in (A) represent the magnitude of the cumulative CO_2_ emission changes comparing the same period between 2019 and 2020. Each dot in (B) represents a province in mainland China, which is plotted on the basis of the share of industrial GDP in provincial total GDP along the *x* axis and the changes in cumulative CO_2_ emissions along the *y* axis. The color of each dot represents the number of days when each province retained its public health response system to the COVID-19 emergency at the top level. Additional lines point to the provinces of Beijing, Tianjin, Hebei, Shanghai, Hubei, and Guangxi. (**C**) and (**D**) are similar to (A) and (B), respectively, but present results for the period from 8 April to 30 April.

Half of China’s provinces are estimated to have reduced their cumulative CO_2_ emissions by more than 15% from 23 January 2020 to 7 April 2020 compared to the same period in 2019 (Fig. 4, A and B). Because the transportation sector has a particularly low CO_2_/NO*_x_* emission ratio and the industrial sector was the major driver of the CO_2_ emission decline, provinces with larger shares of the industrial economy experienced more CO_2_ emission reductions than others (Fig. 4B). The provinces of Jiangsu and Anhui that have the largest industrial economies both reduced their CO_2_ emissions by more than 30%. We also observe large CO_2_ reductions in the Hubei province whose capital is Wuhan and in Beijing and Shanghai, substantially higher than the other provinces with similar economic structures due to the stringent virus control measures in these provinces. Note that the CO_2_ emissions from Guangxi province located in the southwest of China are estimated to have increased by 5% during the lockdown period. This is because the generation of hydroelectric power from January to March in 2020 was 27% lower than that in 2019 due to the severe drought in this region, which has caused an increase in the generation of thermal power by 16% (http://www.stats.gov.cn/). The drought-induced increased fossil fuel use in power plants offset the emission decrease due to lockdown.

With China’s industry slowly recovering from the coronavirus, CO_2_ emissions from most of China’s eastern provinces have returned to their pre–COVID-19 levels in January by April 2020, which are higher than the emissions in the same period of 2019 (Fig. 4, C and D). The provinces whose economy is dominated by industry restarted emissions rapidly, mirroring their larger drop during the lockdown. The provinces that had experienced stringent lockdown did not rebound their CO_2_ emissions substantially, such as Hubei, Hebei, and Tianjin, although these provinces were dominated by the industrial economy. These provinces stayed at the highest emergency response levels against the coronavirus for more than 90 days. The emissions from Beijing and Shanghai were 17 and 22%, respectively, lower than that in 2019 at this period, which exhibited comparable and even larger reductions in CO_2_ emissions compared to the previous 2 months. The Guangxi province, still suffering from the drought in April 2020, increased CO_2_ emissions by 29% compared to those in 2019, corresponding to the 30% increase in thermal power generation.

## DISCUSSION

This study presents the first-ever estimates of the 10-day moving average CO_2_ emissions from satellite observations, derived from the near real-time and high spatial-temporal resolution NO_2_ retrievals from TROPOMI, state-of-the-art chemical transport model GEOS-Chem, and CO_2_-to-NO*_x_* source emission ratios from a detailed inventory of Chinese emissions, MEIC. The substantial short-term variability in emissions due to the COVID-19 lockdown creates an unmistakable signal despite known model uncertainties and satellite observation errors, providing a unique opportunity to develop and validate satellite-based carbon emissions monitoring.

The uncertainties in our results lie in satellite observations of NO_2_ column densities, determination of the NO_2_ column response to surface NO*_x_* emission changes by the GEOS-Chem model, differentiating sectoral NO*_x_* emissions from satellite-constrained estimates, and CO_2_-to-NO*_x_* emission ratios. Using spatial-temporal average and the relative difference of TROPOMI data is expected to cancel a major part of the systematic errors in satellite observations. The major source of uncertainty in this study could be the assumption of locality between NO_2_ columns and NO*_x_* emissions in each grid cell (0.5° × 0.625°) when estimating β. This uncertainty does not affect total NO*_x_* emission estimates according to our sensitivity simulation using the coarse-resolution (2° × 2.5°) global GEOS-Chem model (see Methods), but it can introduce substantial errors in sectoral CO_2_ emission constraints at the grid scale, especially if different source sectors dominate NO*_x_* emissions from adjacent grid cells. In this work, we presented sectoral CO_2_ emissions at an aggregated level (by sector and province) to reduce the uncertainties at grid scale. Corrections on sectoral emissions are also tested through different definitions of the grid cell–dominant source sector, and the inversion results of 10-day mean CO_2_ emissions are found to be stable (fig. S13). The influence of CO_2_-to-NO*_x_* ratios on the estimates of CO_2_ relative reductions from the 2019 MEIC data to the 2020 inversions is marginal, as we use the consistent sector-specific ratio maps with MEIC. The robustness of our estimates could also be demonstrated by the broad consistency of our estimates with previous studies (*6*, *13*) and with independent economic and industrial statistics data collected on the monthly scales (fig. S14). More details about the uncertainty analysis could be found in Methods.

Our findings show that although the COVID-19 lockdown markedly reduced China’s CO_2_ emissions during the first 2 months after the virus outbreak, the emissions rebounded quickly as lockdown measures were lifted. In the first month after Wuhan reopened, which essentially marked the end of the country lockdown, China’s CO_2_ emissions again reached pre–COVID-19 levels at the beginning of January 2020 and exceeded the level of emissions in the same period of 2019. These trends in emissions were strongly influenced by the industry sector, which was the main driver in both the decline and rebound of Chinese CO_2_ emissions over the short period. It is difficult to predict the long-term effect of the pandemic on energy use and thus CO_2_ emissions. Lockdowns in some other regions are now being relaxed, and economic stimulus packages are now being implemented in China and other countries affected by COVID-19. But our results show that the effects on energy and emissions depend on the strictness and duration of lockdowns in different regions, the economic structures and recovery stimulus programs of those regions, the effectiveness of such stimulus, and also whether and where a second wave of the virus and lockdowns recur. Regardless, the method we have developed and demonstrated here will support efforts to monitor changes in energy systems and emissions in near real time as we navigate into that uncertain future. This approach is potentially applicable to other global regions where anthropogenic NO*_x_* emissions are dominant and evident declines in NO_2_ columns are observed during COVID-19 lockdown, such as India, western Europe, and the United States (*5*).

## METHODS

### Integrated model framework

We develop an integrated model framework combining both top-down and bottom-up information to estimate 10-day moving average NO*_x_* and CO_2_ emissions in China from January to April in 2020. This framework includes four processing steps. First, we make a preliminary estimate of China’s daily NO*_x_* and CO_2_ emissions in 2020 using the bottom-up emission model MEIC. Then, we develop an inverse modeling system to infer 10-day moving average total NO*_x_* emissions in 2020 from TROPOMI NO_2_ column retrievals. Next, we use the inversion estimated NO*_x_* emissions combined with the bottom-up estimated emission maps to constrain sectoral NO*_x_* emission dynamics. Last, the TROPOMI-constrained sectoral NO*_x_* emissions are transformed into sectoral CO_2_ emissions through sector-specific CO_2_/NO*_x_* emission ratio maps. The four steps are described in the following sections, and the methodological framework is presented in fig. S1.

### Bottom-up daily emission estimates in 2020

We make a preliminary estimate of China’s daily NO*_x_* and CO_2_ emissions in 2020 using the MEIC model (*28*). The MEIC model is briefly described in the Supplementary Materials, and more details can be found in the cited papers within the text. We combine the newly developed 2019 emissions (fig. S2) in MEIC with the relative changes in activity data and emission factors from 2019 to 2020 to update China’s emissions to 2020, which is similar to the method in (*13*).

### Monthly emission estimates in 2020

The 2019 emissions in MEIC are updated to 2020 based on the year-on-year change in monthly activity data and emission factor of each source. Scaling factors calculated between the level in a given month of 2020 and that in the same month of 2019 are used to extrapolate the 2019 emissions to 2020. NO*_x_* emission factors in MEIC slightly declined from the beginning to the end of 2019 due to NO*_x_* pollution control, while CO_2_ emission factors are assumed unchanged. Multiple data sources are used to predict the activity change of different sources as below.

#### Power and industry sectors

Monthly growth rates of electricity generation, cement production, iron production, and industrial GDP from 2019 to 2020 (http://www.stats.gov.cn/) are used to represent activity changes in power plants, cement plants, iron and steel plants, and the other industries, respectively. Several assumptions are needed for the emission estimates in 2020. First, the statistics data for January and February were combined, so activity changes in these 2 months are assumed the same. Second, the growth rate in the electricity generation includes coal-, oil-, and natural gas–fired electricity together that cannot be further separated. We assume that the activity data of all of the thermal power plants follow the same monthly changes from 2019 to 2020. Third, the industrial GDP is used to roughly represent the relative change of the industrial activities, although the economic values may not directly reflect the changes in the fossil fuel burned. These three assumptions mentioned above could cause uncertainties in the estimates of monthly emissions in the power and industrial sectors in 2020.

#### Residential sector

We calculate the population-weighted HDD for all of China’s cities in each month of 2019 and 2020 to represent the relative change of the residential energy use in each city. The gridded population data are exploited from Gridded Population of the World, Version 4 (GPWv4) (*32*), and the surface temperature data are derived from the ERA5 dataset produced by European Centre for Medium-Range Weather Forecasts (ECMWF) (*33*). The reference temperature is set to 18°C (*34*).

#### Transport sector

We use the Baidu migration data (https://qianxi.baidu.com/2020/) within each city to represent the relative change in activity data of the transport sector from 2019 to 2020 in each city. There could be uncertainties in using Baidu migration data to estimate transport emissions because the Baidu location–based services track all of the surface transport within cities including walk, bicycles, vehicles, and subways. The way we use this index to predict activity changes in both the road and nonroad sources could involve uncertainties.

### Daily emission estimates in 2020

The estimated monthly emissions in 2020 are allocated into daily time scales based on daily temporal profiles. The power plant emissions are split into daily scales based on the daily coal consumption of the six major power generation groups in China (fig. S3A), which accounted for 12% of China’s coal consumption in the power sector in 2019. The industrial emissions from iron and steel plants, coke plants, and coal mines are split into daily scales based on the daily operating rates (fig. S3, B to E). All of the other industrial emissions are split into a daily scale based on the daily coal consumption in the major power generation groups (fig. S3A). The monthly emissions from the residential and transport sectors are split into daily emissions using the daily variation of population-weighted HDD (fig. S4) and the Baidu index (fig. S5) in each city.

### Total NO*_x_* emissions inferred from TROPOMI in 2020

We then use a nested-grid GEOS-Chem model (*30*) at the horizontal resolution of 0.5° × 0.625° to establish the spatial-temporal varied local relationship between the changes in surface NO*_x_* emissions and changes in NO_2_ tropospheric vertical column densities (TVCDs). Such a relationship is applied to changes in the satellite-observed NO_2_ TVCDs from 2019 to 2020 derived from the TROPOMI (*19*) to infer total NO*_x_* emissions in 2020 on the 10-day moving average scale. Changes in NO_2_ TVCDs due to meteorological variations have been accounted for and removed.

### NO*_x_* emissions estimated from TROPOMI

Using TROPOMI observations over regions dominated by anthropogenic sources (*3*), we infer China’s anthropogenic NO*_x_* emissions of 10-day mean in 2020 using the following equation

$$E_{t,i,\text{TROPOMI},2020}=(1+\beta_{t,i}{\left(\frac{∆\Omega }{\Omega }\right)}_{t,i,\text{anth}})\times E_{t,i,\text{bottom}-\text{up},2019}$$ (1)

where *i* represents a model grid cell (0.5° × 0.625°), *t* represents a 10-day time window, *E*_*t,i,*TROPOMI,2020_ is the total NO*_x_* emission in 2020 inferred from TROPOMI NO_2_ TVCDs, *E*_*t,i,*bottom-up,2019_ is the total NO*_x_* emission in 2019 estimated by the MEIC emission model, β*_t,i_* is a unitless factor that represents the local sensitivity of changes in NO_2_ TVCDs to changes in anthropogenic NO*_x_* emissions in each model grid cell, and (∆Ω/Ω)_*t,i,*anth_ is the relative change in NO_2_ TVCDs from 2019 to 2020 due to anthropogenic emission changes.

### Simulation of β based on GEOS-Chem

β*_t,i_* is estimated using the method from (*29*) with the GEOS-Chem model on the basis of the assumption of collocation between NO_2_ columns and NO*_x_* emissions in each model grid cell. We use the GEOS-Chem version 12.3.0 (https://doi.org/10.5281/zenodo.2620535) driven by assimilated meteorological fields from the NASA Global Modeling and Assimilation Office’s Modern-Era Retrospective analysis for Research and Applications Version 2 (MERRA-2) (*35*). For the simulation over China, we use the nested-grid configuration over Southeast Asia at a spatial resolution of 0.5° × 0.625° (*30*), with boundary conditions adopted from a 2° × 2.5° global simulation. We use the “tropchem” mechanism that simulates full chemistry in the troposphere. Vertical mixing in the planetary boundary layer is simulated using a nonlocal mixing scheme (*36*).

We first do a baseline simulation in 2019. Anthropogenic emissions over Southeast Asia are taken from the MIX inventory (*37*), while emissions in mainland China are replaced by emissions from the MEIC model for the year 2019. The simulation also includes additional NO*_x_* emission sources such as lightning (*38*), soil and fertilizer (*39*), shipping, and aircraft. Figure S6 shows the comparison between coincidently sampled simulated and TROPOMI NO_2_ TVCDs for the baseline simulation in 2019. The spatial pattern of the baseline simulation is consistent with the NO_2_ TVCDs from TROPOMI, and they agree very well with *R*^2^ = 0.89 and slope = 0.98. Then, we do another perturbation simulation reducing China’s NO*_x_* emissions by 40%. These two simulations both use meteorological fields in 2019. β*_t,i_* is then calculated by the following equation

$$\beta_{t,i}=\frac{{∆E}_{t,i,\text{bottom}-\text{up},2019}}{E_{t,i,\text{bottom}-\text{up},2019}}÷\frac{\Omega_{t,i,\text{perturbed},2019}-\Omega_{t,i,\text{base},2019}}{\Omega_{t,i,\text{base},2019}}$$ (2)

where perturbed and base represent the perturbation and baseline simulation, respectively. Ω_*t,i,*perturbed,2019_ and Ω_*t,i,*base,2019_ are GEOS-Chem simulated NO_2_ TVCDs at the TROPOMI overpass time. ∆*E*_*t,i,*bottom-up,2019_/*E*_*t,i,*bottom-up,2019_ is the 40% reduction in anthropogenic NO*_x_* emissions.

Figure S7 shows the spatial variation of 4-month averaged β coincidently sampled with the TROPOMI data. β tends to be less than one in polluted regions such as the North China Plain, the Yangtze River Delta, and the Pearl River Delta, because an increase in NO*_x_* emissions consumes OH and increases the NO*_x_* lifetime. While in clean areas where an increase in NO*_x_* emissions decreases the NO*_x_* lifetime, β tends to be greater than one. Figure S7B shows the 10-day moving average of national β over China. β is smaller in winter when the concentrations of OH and RO_2_ radicals are lower and increases in spring, which reflects a longer NO*_x_* lifetime in winter time. We further tested the sensitivity of β*_t,i_* to the grid resolution and the perturbation of emissions by changing the GEOS-Chem model resolution to 2° × 2.5° and through several sensitivity simulations in the Supplementary Materials.

### Estimation of NO_2_ TVCD changes excluding meteorological impacts

(∆Ω/Ω)_*t,i,*anth_ are derived from TROPOMI NO_2_ TVCD changes from 2019 to 2020 with the help of the GEOS-Chem model quantifying the contributions of anthropogenic emission change. We conduct a simulation with China’s anthropogenic emissions fixed in 2019 driven by meteorological fields in 2020. The influences of other factors other than China’s anthropogenic emissions on NO_2_ TVCDs are quantified by comparing the anthropogenic emission fixed simulation with Ω_*t,i,*base,2019_ driven by emissions and meteorological fields in 2019. Then, we get

$${\left(\frac{∆\Omega }{\Omega }\right)}_{t,i,\text{anth}}=\frac{\Omega_{t,i,\text{sate},2020}}{\Omega_{t,i,\text{sate},2019}}-\frac{\Omega_{t,i,\text{fixemis},2020}}{\Omega_{t,i,\text{base},2019}}$$ (3)

where sate and fixemis represent satellite observation and anthropogenic emission fixed simulation, respectively. Ω_*t,i,*fixemis,2020_ is GEOS-Chem simulated NO_2_ TVCDs at the TROPOMI overpass time from the simulation with fixed anthropogenic emissions. Ω_*t,i,*sate,2019_ and Ω_*t,i,*sate,2020_ are TROPOMI NO_2_ TVCDs in 2019 and 2020, respectively.

We use NO_2_ TVCDs from the official TM5-MP-DOMINO version 1.2/1.3 offline product (http://www.temis.nl/airpollution/no2col/data/tropomi/) (*19*). Only pixels with quality assurance value above 0.5 and cloud fraction below 30% are kept to reduce the retrieval errors. We calculate the 10-day moving average (fig. S8) to smooth out daily fluctuations in NO_2_ TVCDs caused by random errors and increase the sample number and the spatial coverage. The grid cells dominated by natural sources [defined as NO_2_ TVCDs below 1 × 10^15^ molecules/cm^2^ according to (*3*)], which account for more than half of grid cells in China (fig. S9), are excluded in our study. In the remaining grid cells, the average share of grid cells with more than five valid sample days reaches 87%, and they cover 81% of anthropogenic NO*_x_* emissions over China (fig. S9), indicating that the 10-day moving average values used here are representative for NO*_x_* emissions over China.

### Sectoral NO*_x_* emissions constrained by TROPOMI in 2020

Because the CO_2_-to-NO*_x_* emission ratio varies by source sector, it is important to know sectoral NO*_x_* emissions to transform NO*_x_* emissions into CO_2_ emissions. Our bottom-up method provides sectoral emissions at the daily scales in 2020, while the imperfect daily statistics data cause uncertainties in bottom-up sectoral emissions. Here, we use the total NO*_x_* emission inferred from TROPOMI to constrain bottom-up estimated NO*_x_* emission maps in 2020 by source sector, based on the emission differences revealed by the model grid cells dominated by a single source sector.

The sector-specific scaling factors to correct the preliminary bottom-up NO*_x_* emissions are calculated using the following equation

$${\text{scalefactor}}_{t,s}=1+\frac{\Sigma_{i}(E_{t,i,\text{TROPOMI},2020}^{s}-E_{t,i,\text{bottom}-\text{up},2020}^{s})}{\Sigma_{i}(E_{t,i,\text{bottom}-\text{up},2020}^{s})}$$ (4)

where $E_{t,i,\text{TROPOMI},2020}^{s}$ and $E_{t,i,\text{bottom}-\text{up},2020}^{s}$ are TROPOMI-constrained and bottom-up estimated NO*_x_* emissions on grid cell *i* that are dominated by source sector *s* (i.e., power, industry, residential, and transport) in time *t*, respectively. scalefactor_*t*, *s*_ is the scaling factor used to correct the bottom-up estimated NO*_x_* emissions from sector *s* in time *t*.

The dominant emission source sector in each grid cell *i* is defined as the sector that accounts for more than 50% of NO*_x_* emissions in each grid. Before the COVID-19 lockdown on 23 January 2020, we assume that the dominant emission source sector in each model grid in 2020 is consistent with that at the same time of 2019, which is estimated on the basis of the MEIC emissions in January 2019. After 23 January 2020, because the COVID-19 lockdown has influenced the relative contribution of emission sources, we use the corrected bottom-up sectoral emission map on the prior day to identify the dominant emission source sector on each day. Figure S10 shows the examples of grid cells dominated by power, industry, and transport sectors, where the TROPOMI observes large NO_2_ column enhancement at the locations of point sources and road networks. In general, the grid cells with a dominant emission source sector contribute 20 to 50% of NO*_x_* emissions from that sector in China’s national totals (fig. S11C).

Then, the relative differences between TROPOMI-constrained and bottom-up estimated NO*_x_* emissions over grid cells dominated by each sector are calculated to derive the scaling factors for each sector, as shown in Eq. 4. Figure S11 shows the differences in NO*_x_* emissions between the TROPOMI-based and bottom-up estimates over the grid cells dominated by different source sectors. The bottom-up estimates overestimate industrial and transport emissions during the lockdown, especially at the beginning of lockdown, but slightly underestimate emissions after the end of lockdown. This is due to the imperfect knowledge of the daily dynamics of the transport and the industrial activities, and the proxies such as the people migration index and the industrial GDP are not accurate enough to predict emissions. The power sector shows smaller uncertainties because we have monthly electricity generation and the daily coal use in the power plants that account for 12% of China’s power sector coal consumption. However, because of the uncertainties in these data as discussed before, the power sector emissions are still slightly underestimated in the bottom-up inventory.

Last, the bottom-up estimated NO*_x_* emissions are corrected by sector based on the following equation

$$E_{s,t,i,\text{bottom}-\text{up},\text{corrected},2020}=E_{s,t,i,\text{bottom}-\text{up},2020}\times {\text{scalefactor}}_{t,s}$$ (5)

where *E*_*s*,*t*,*i*,bottom−up,corrected,2020_ and *E*_*s*,*t*,*i*,bottom−up,2020_ represent the corrected and preliminary bottom-up NO*_x_* emissions from sector *s* in time *t*, respectively. Then, we scale the corrected bottom-up emissions to be consistent with the TROPOMI-based NO*_x_* emissions to reduce the remaining differences. The following equation is used

$$E_{s,t,i,\text{constrained},2020}=E_{s,t,i,\text{bottom}-\text{up},\text{corrected},2020}\times \frac{E_{t,i,\text{TROPOMI},2020}}{\Sigma_{s}E_{s,t,i,\text{bottom}-\text{up},\text{corrected},2020}}$$ (6)

where *E*_*s*, *t*, *i*, constrained,2020_ represents the sectoral NO*_x_* emissions constrained by TROPOMI.

### Sectoral CO_2_ emissions constrained by TROPOMI in 2020

Sectoral CO_2_ emissions are estimated from the TROPOMI-constrained sectoral NO*_x_* emissions based on the sector-specific ratios of CO_2_ to NO*_x_* emissions using the following equation

$$\begin{array}{c} C_{s,t,i,\text{constrained},2020}=E_{s,t,i,\text{constrained},2020}\times \\ \frac{\text{EFCO}2_{s,i,\text{bottom}-\text{up},2019}}{\text{EFNO}x_{s,i,\text{bottom}-\text{up},2019}\times (1-{r\text{NO}x}_{s,i})} \end{array}$$ (7)

where *C*_*s*, *t*, *i*, constrained,2020_ represents sectoral CO_2_ emissions from sector *s* on grid cell *i* in time *t*, EFCO2_*s*, *i*, *b*ottom − up,2019_ and EFNO*x*_*s*, *i*, bottom − up,2019_ represent the emission factors of CO_2_ and NO*_x_* of sector *s* on grid cell *i* derived from the MEIC emission data averaged from January to April in 2019, and *r*NO*x_s,i_* represents the reduction ratio of NO*_x_* emission factor from 2019 to 2020, which is assumed equivalent to the reduction from the beginning to the end of 2019. The MEIC emission model estimates that the NO*_x_* emission factors of the power and cement plants have declined by 6 to 10% in 2019 due to the pollution control.

MEIC estimates substantially varied CO_2_-to-NO_x_ emission ratios by source due to different combustion and pollution control techniques used in each source. Power, industry, residential, and transport sectors have CO_2_-to-NO_x_ emission ratios of 979, 623, 917, and 141 g CO_2_/g NO_2_, on average, in 2019. The largest value for the power sector is due to pollution controls on NO*_x_*, which reduces NO*_x_* emissions substantially. The large value for the residential sector is because the residential stoves generate fewer NO*_x_* emissions per fuel burnt than the high-efficiency boilers in the power and industry sectors. The low CO_2_-to-NO*_x_* emission ratio of the transport sector is due to the low carbon content of petroleum fuels and the high emission factors of NO*_x_* from diesel vehicles and nonroad equipment. The daily variability of emission contributions from different sectors drives the daily evolution of China’s average CO_2_-to-NO*_x_* emission ratio (fig. S12).

The CO_2_-to-NO*_x_* emission ratios are different in 2020 compared to 2019 due to the influence of COVID-19 lockdown (fig. S12). The largest difference is that the CO_2_-to-NO*_x_* emission ratio slightly decreased during the Spring Festival holiday in 2019 but increased over the same period in 2020. In normal years like 2019, the decrease of CO_2_-to-NO*_x_* emission ratio during the Spring Festival holiday was driven by the reduced power and industrial emissions, while the transport emissions changed much less. However, the transport emissions decreased substantially during the Spring Festival holiday in 2020 due to lockdown measures. The CO_2_-to-NO*_x_* emission ratios are much lower in the transport sector than in the other sectors, so the average CO_2_-to-NO*_x_* emission ratios are estimated to have increased during the lockdown in 2020. After the lockdown measures eased, the average CO_2_-to-NO*_x_* emission ratios in 2020 returned to the same levels as in 2019.

### Uncertainties

Our results are subject to several uncertainties and limitations, including errors in the satellite NO_2_ TVCDs, uncertainties in β, the dynamic sectoral distribution of TROPOMI-constrained NO*_x_* emissions, and the CO_2_-to-NO*_x_* emission ratios used to convert NO*_x_* emissions to CO_2_ emissions. The uncertainty in each step of our analysis is discussed below.

First, the satellite retrievals of NO_2_ TVCDs usually suffer from uncertainties in the radiative transfer model and in the ancillary data used for calculating the stratospheric NO_2_ background and the air mass factors. For example, the TROPOMI single-pixel errors are typically ~40 to 60% in winter time (*5*). We use spatial and temporal averaging in our analysis to reduce random errors. The averaging approach also helps reduce part of the systematic errors in the air mass factor, i.e., those originating from a priori NO_2_ profiles and surface albedo assumptions. Meanwhile, TROPOMI NO_2_ is found to be systematically underestimated over China (*40*), especially over polluted regions. However, relative differences between 2019 and 2020 are used to derive NO*_x_* emission changes, which is expected to cancel out a major part of the systematic errors.

Second, the β value simulated by the GEOS-Chem model reflects the feedback of NO*_x_* emissions on NO*_x_* chemistry, which could be affected by many factors. Particularly, the assumption of locality between NO_2_ columns and NO*_x_* emissions may introduce substantial uncertainties when estimating β (Eq. 2) and NO*_x_* emissions (Eq. 1) in winter at the model resolution (0.5° × 0.625°) because NO_2_ can transport from neighboring grids (*41*). In turn, this will further affect the sectoral CO_2_ emission estimates when different sectors dominate emissions in adjacent model grid cells. We estimated β and NO*_x_* emissions by using the 2° × 2.5° GEOS-Chem model and found that the resulting differences in NO*_x_* emission estimates are within 3%, indicating that the NO*_x_* emission estimates are not largely affected by model resolution. To diminish the errors in sectoral CO_2_ emission estimates at the grid level, we presented and discussed sectoral CO_2_ emissions at an aggregated level (national total or by province) in the main text. The β value could also be affected by the model representation of chemical and physical processes (e.g., transport and deposition) and changes in emissions of other species involved in the NO*_x_* chemistry. We evaluate the model simulation against satellite data to prove the model’s ability to capture the characteristics of NO_2_ columns. We also conduct several emission perturbation scenario simulations and found that the β value is not sensitive to the magnitude of emission perturbations (see the Supplementary Materials).

Third, because the CO_2_-to-NO*_x_* ratio is sector specific, we need to capture the dynamic sectoral distribution of NO*_x_* emissions from January to April in 2020 for the conversion from NO*_x_* to CO_2_. We use the TROPOMI-constrained NO*_x_* emissions to correct the sectoral emissions from the bottom-up estimates, based on the grid cells dominated by one single emission source, as discussed above. The thresholds used for the determination of dominant source sector in each grid could be the largest source of error here. Sensitivity tests using threshold values from 0.5 to 0.8 obtain similar CO_2_ emission estimations (fig. S13), reflecting the robustness of our method.

Last, the sector-specific CO_2_-to-NO*_x_* ratio maps that we achieved from the MEIC emission model also include some uncertainties, which could influence the absolute magnitude of the CO_2_ emission estimates. The uncertainties in NO*_x_* emission inventories are typically higher than those in CO_2_ emission inventories, while the MEIC NO*_x_* inventory that we used in this study has revealed a good performance when modeling with the GEOS-Chem model and comparing it to the TROPOMI NO_2_ column observations (fig. S6). Besides, the influence of the potential uncertainties in the CO_2_-to-NO*_x_* ratios on the estimates of CO_2_ emission reductions from 2019 to 2020 is relatively marginal, because we use consistent sector-specific CO_2_-to-NO*_x_* ratio maps with the MEIC model and the comparison is also performed with the MEIC emissions.

We also compare the CO_2_ emissions estimated from the TROPOMI-constrained NO*_x_* emissions with the economic and industrial statistics data collected on the monthly scales (fig. S14). The relative changes in the 2020 CO_2_ emissions compared to those in 2019 are consistent with those indicators of the industry in China, which independently evaluates our estimates of the CO_2_ emission changes.

## Supplementary Material

http://advances.sciencemag.org/cgi/content/full/6/49/eabd4998/DC1

Adobe PDF - abd4998_SM.pdf

Satellite-based estimates of decline and rebound in China’s CO2 emisions during COVID-19 pandemic

## SUPPLEMENTARY MATERIALS

Supplementary material for this article is available at http://advances.sciencemag.org/cgi/content/full/6/49/eabd4998/DC1
