# Supplementary material for: Satellite-based estimates of decline and rebound in China’s CO2 emissions during COVID-19 pandemic
Source: Sci Adv. 2020 Dec 2;6(49):eabd4998. doi: 10.1126/sciadv.abd4998 (PMC7821878; doi:10.1126/sciadv.abd4998)
Supplement: http://advances.sciencemag.org/cgi/content/full/6/49/eabd4998/DC1 [file supp_6_49_eabd4998__index.html]

Science Advances | Science AdvancesAAASSearchScience AdvancesMenu

## Supplementary Materials

# Satellite-based estimates of decline and rebound in China’s CO2 emissions during COVID-19 pandemic

Bo Zheng, Guannan Geng, Philippe Ciais, Steven J. Davis, Randall V. Martin, Jun Meng, Nana Wu, Frederic Chevallier, Gregoire Broquet, Folkert Boersma, Ronald van der A, Jintai Lin, Dabo Guan, Yu Lei, Kebin He, Qiang Zhang

Download Supplement

**This PDF file includes:**

- MEIC emission model
- Sensitivity simulations of β value
- Figs. S1 to S14
- Table S1
- References

**Files in this Data Supplement:**

- Adobe PDF - abd4998\_SM.pdf
