# Supplementary material for: Satellite-based estimates of decline and rebound in China’s CO2 emissions during COVID-19 pandemic
Source: Sci Adv. 2020 Dec 2;6(49):eabd4998. doi: 10.1126/sciadv.abd4998 (PMC7821878; doi:10.1126/sciadv.abd4998)
Supplement: http://advances.sciencemag.org/cgi/content/full/6/49/eabd4998/DC1 [file supp_6_49_eabd4998__1.pdf]

Science Advances
AAAS

advances.sciencemag.org/cgi/content/full/6/49/eabd4998/DC1

Supplementary Materials for

Satellite-based estimates of decline and rebound in China's CO_2 emissions during COVID-19 pandemic

Bo Zheng, Guannan Geng, Philippe Ciais, Steven J. Davis, Randall V. Martin, Jun Meng, Nana Wu, Frederic Chevallier, Gregoire Broquet, Folkert Boersma, Ronald van der A, Jintai Lin, Dabo Guan, Yu Lei, Kebin He, Qiang Zhang*

*Corresponding author. Email: qiangzhang@tsinghua.edu.cn

Published 2 December 2020, *Sci. Adv.* **6**, eabd4998 (2020)
DOI: 10.1126/sciadv.abd4998

This PDF file includes:

MEIC emission model
Sensitivity simulations of β value
Figs. S1 to S14
Table S1
References

Supplementary Materials

MEIC emission model

The MEIC emission model was developed by Tsinghua University (*28, 42*). MEIC tracks the evolution of the manufacturing and pollution control technologies for more than 700 anthropogenic emission sources in China, which can be integrated into five source sectors of power, industry, residential, transportation, and agriculture. The coal-fired power plants in the power sector (4*3, 44*) and the cement plants in the industry sector (*45*), the two important sources of NO_x and CO_2 emissions in China, are treated as point sources with accurate geographic locations, where emissions are estimated using the facility-level activity data and emission factors. The other emission sources are all estimated as area sources using the province- or county-level parameters (*46, 47*). The MEIC emission inventory has an accurate representation of emission spatial distributions (*48, 49*) and emission annual trends (*41, 50*), evaluated by satellite observations. The MEIC model uses monthly and daily temporal profiles including electricity generation, industry factory operating rate, traffic volume index, and heating degree day (i.e., the number of degrees that a day's average temperature is below 18 °C) to disaggregate the annual emission estimates to monthly and daily emissions. The daily sectoral NO_x emissions from January to April in 2019 are shown in Figure S2a. The low values of CO_2 to NO_x emission ratio around the Chinese New Year in 2019 (Figure S2b) are due to reduced activities in power and industry sectors during the holiday.

Sensitivity simulations of β value

The estimation of β has been proved robust at coarse spatial resolutions. Lamsal et al. (*29*) used the global GEOS-Chem model (2° × 2.5°) to perform several tests of β. They find that a perturbation of 30% NO_x emissions instead of 15% changes global averaged β by <2%. Increasing anthropogenic VOCs and CO by 15% increases the global value of β by 2.8% and 1.0%, respectively. When NO_x emissions are only perturbed in a single grid cell in Ohio, β values in neighboring grids are affected by 2–6%, which indicates that β is barely influenced by the transport of NO_2 from neighboring grid cells at the resolution of 2° × 2.5°.

In this study, we use the nested-grid model with a higher spatial resolution (0.5°×0.625°) than the global model used in Lamsal et al. (*29*), which resolves the nonlinear NO_x chemistry and heterogeneous emission sources. However, β obtained from smaller grid cells might be more affected by the transport of NO_2 from neighboring grids or background area, especially during winter time when the NO_2 lifetime is longer. We conduct a sensitivity test using the global GEOS-Chem model to simulate β as a comparison. Results show that the national average value of β is similar to the nested-grid model, and the estimated NO_x emissions are within a 3% difference.

We also find that perturbing NO_x emissions by 30% or 50% instead of 40% only changes β by –0.7% and 0.8%, respectively. We also conduct sensitivity simulations with an additional 20% decrease in CO and a 40% decrease in VOCs, which increase β by 2.5% nationally. The results of the sensitivity tests show that the β value is not sensitive to emission perturbation.

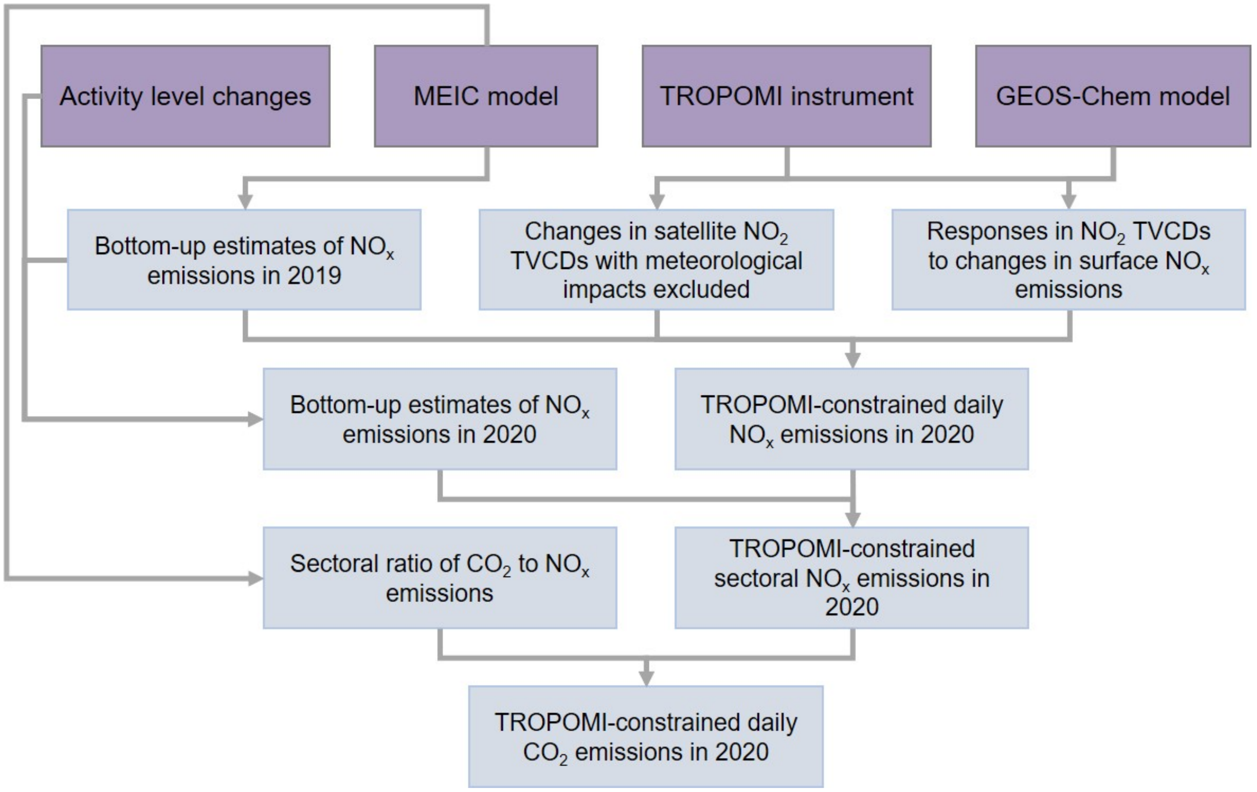

Fig. S1. Methodology framework to infer daily CO_2 emissions from TROPOMI NO_2 TVCDs over China.

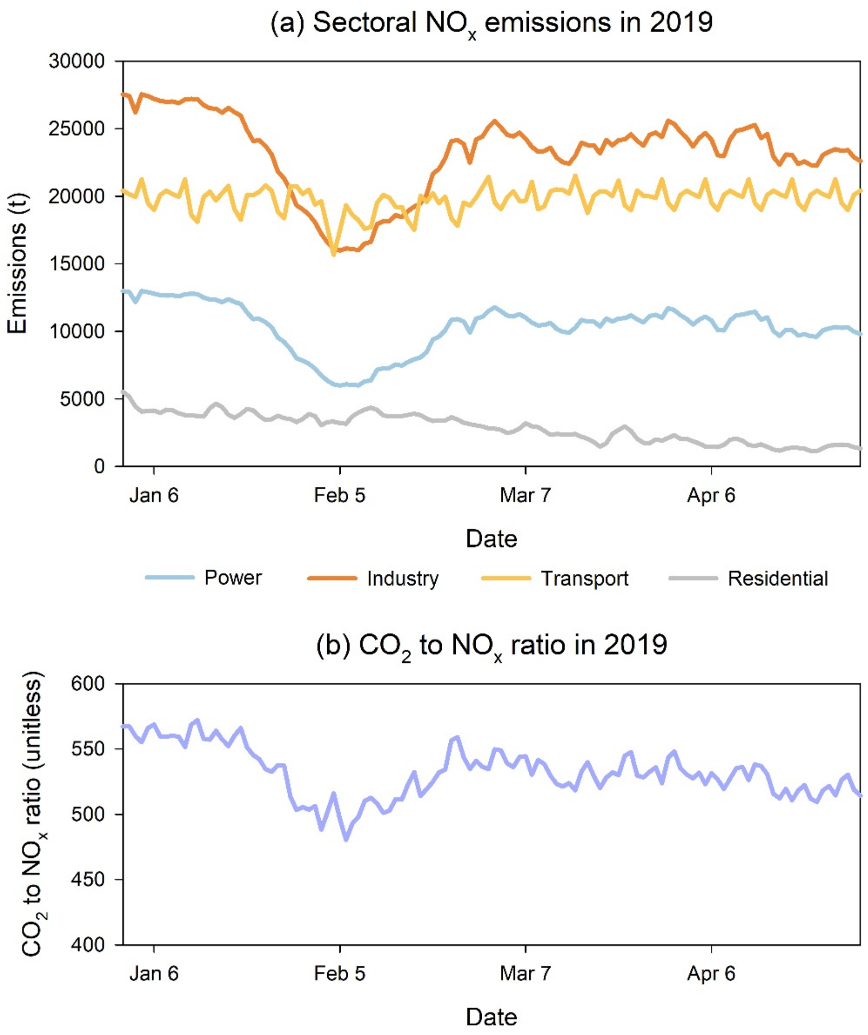

Fig. S2. MEIC daily emissions from January to April in 2019. Fig (**a**) presents the daily sectoral NO_x emissions from the source sectors of power, industry, transport, and residential. Fig (**b**) presents the daily CO_2 to NO_x emission ratio in 2019.

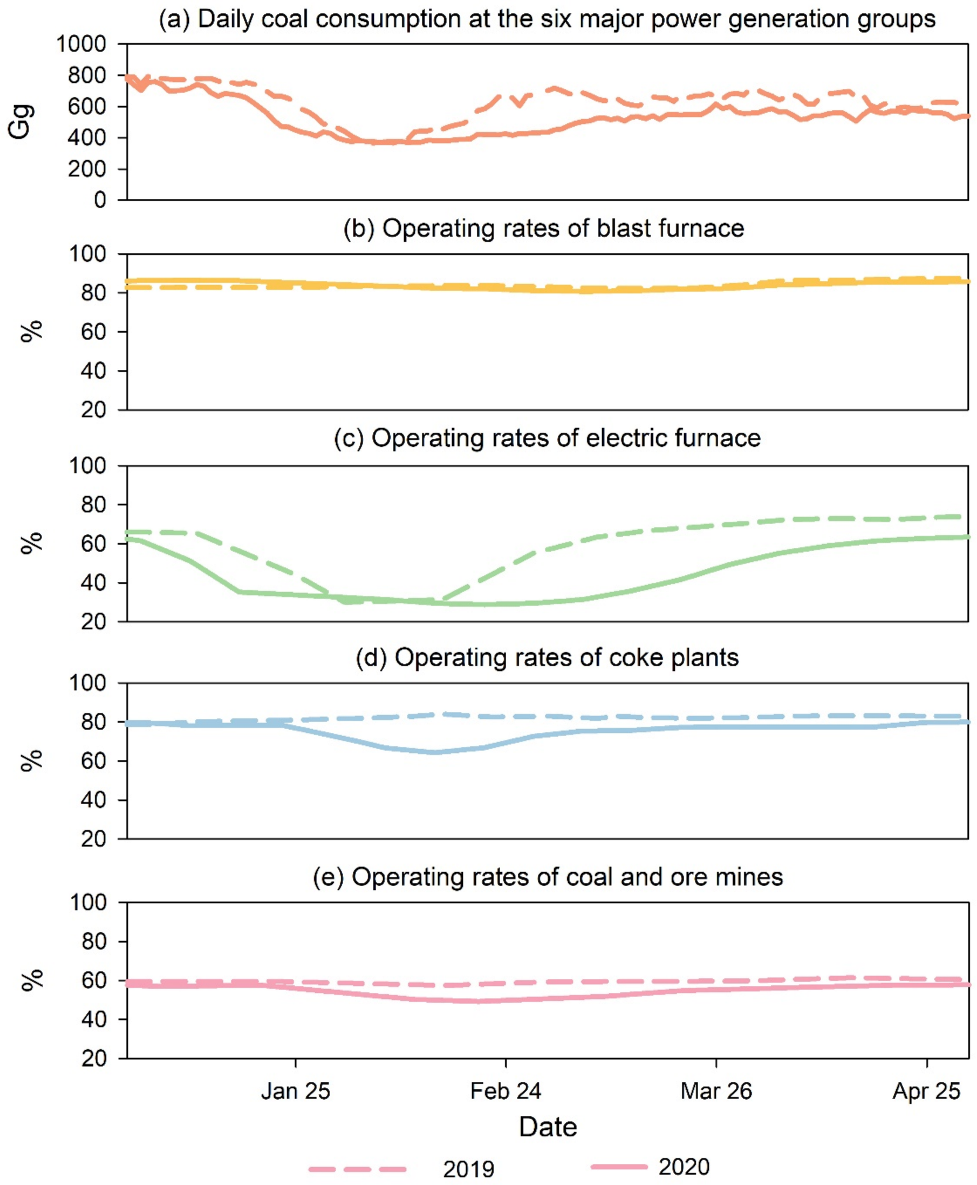

Fig. S3. Daily activities of power and industry sectors from January to April in 2019 and 2020. Fig. (**a**) presents daily coal consumption at the six major power generation groups. Fig. (**b**)-(**e**) present the operating rates of blast furnace, electric furnace, coke plants, and coal and ore mines.

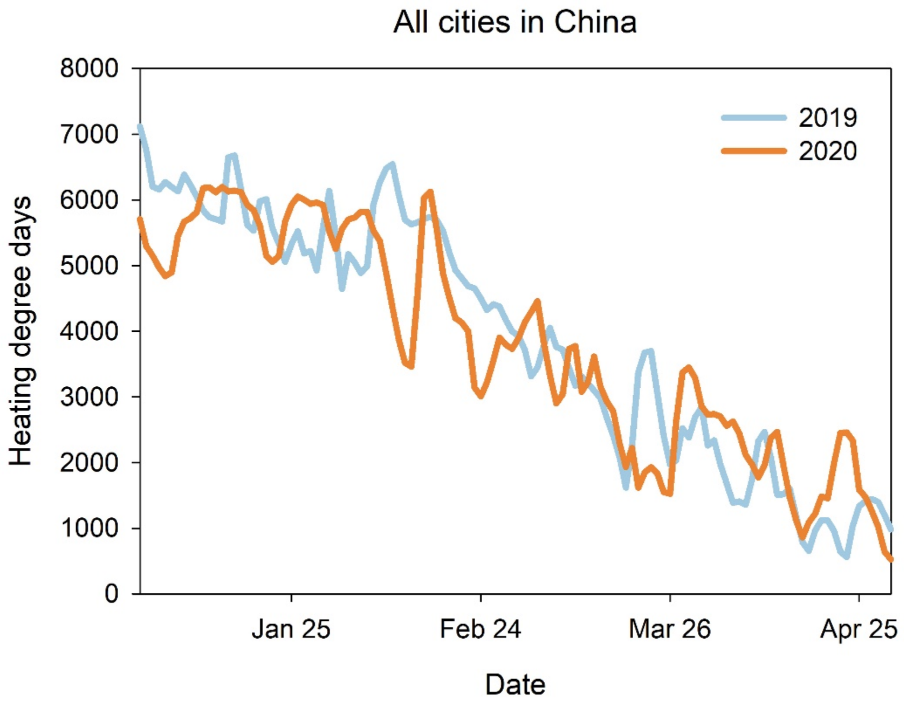

Fig. S4. Population-weighted heating degree days of all cities in China. The data are estimated for each city at a daily scale with the reference temperature of 18℃. The curves represent the sum of the population-weighted heating degree days of all the cities in 2019 and 2020.

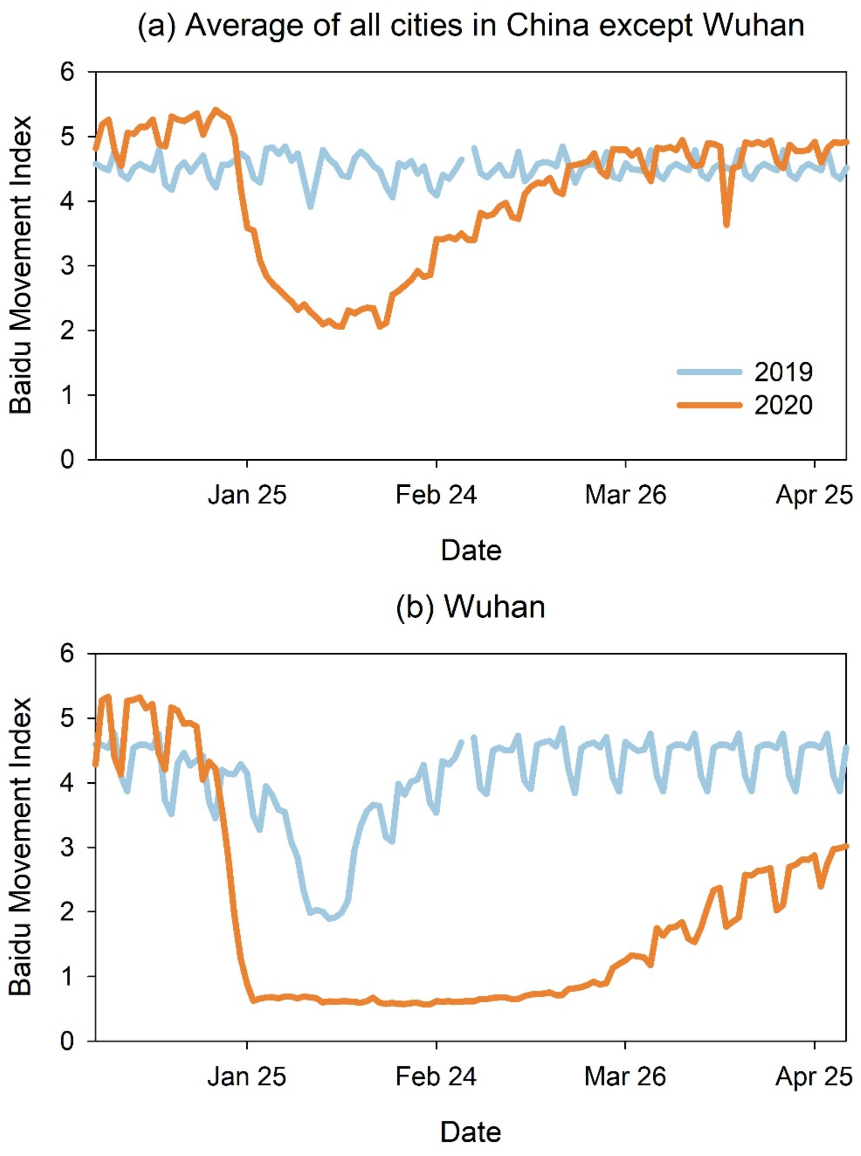

Fig. S5. Daily population migration data within city from the Baidu location-based services. (a) the average of all of China's cities except Wuhan, and (b) Wuhan. The Baidu migration data are derived from the website https://qianxi.baidu.com/2020/.

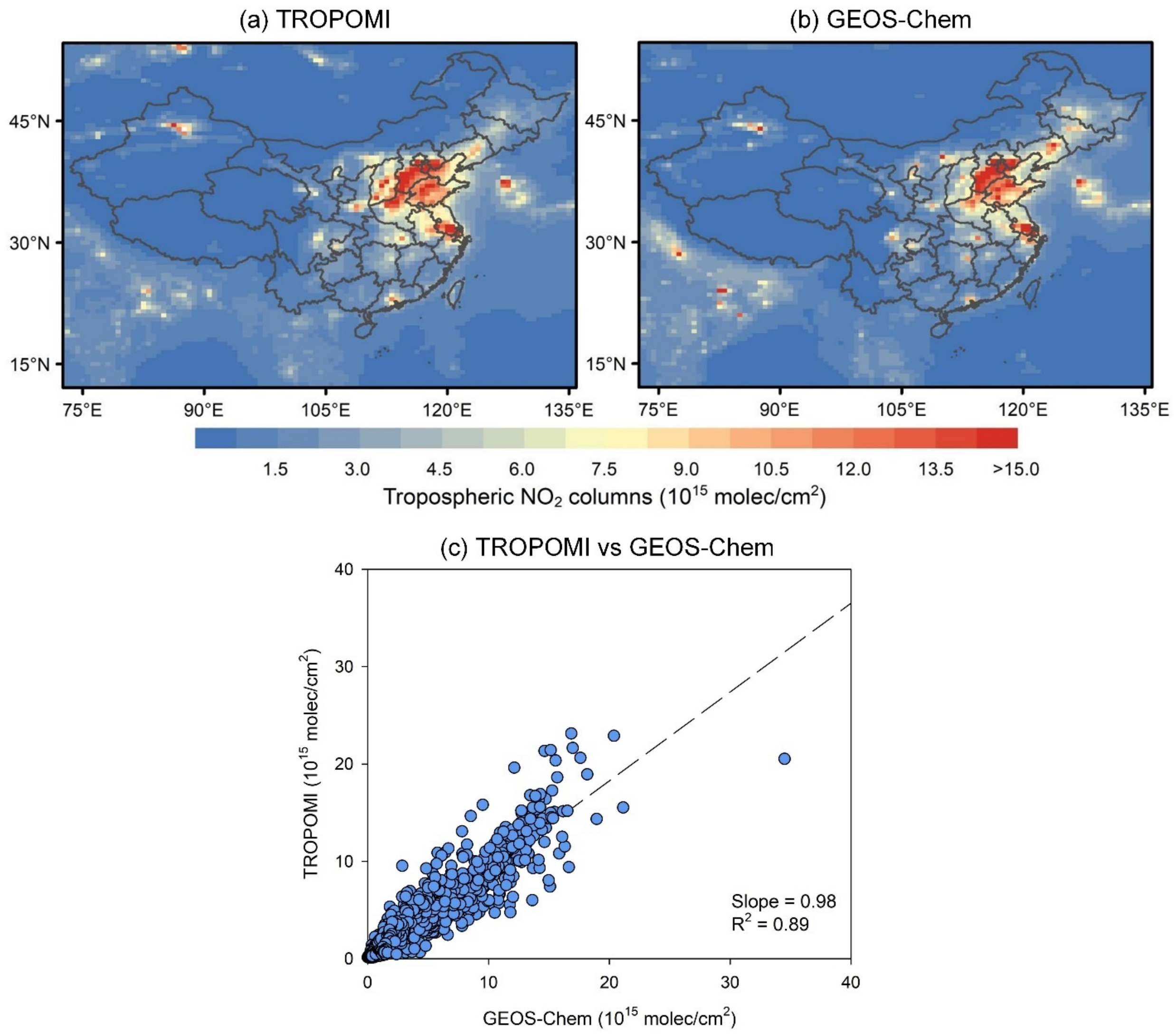

Fig. S6. Evaluation of the GEOS-Chem baseline scenario with NO_2 TVCDs from TROPOMI. Spatial distribution of coincidently sampled four-month averaged NO_2 TVCDs from (a) TROPOMI and (b) GEOS-Chem. (c) Scatter plot between model simulations and TROPOMI retrievals, with regression R^2 and slope displaced in the panel.

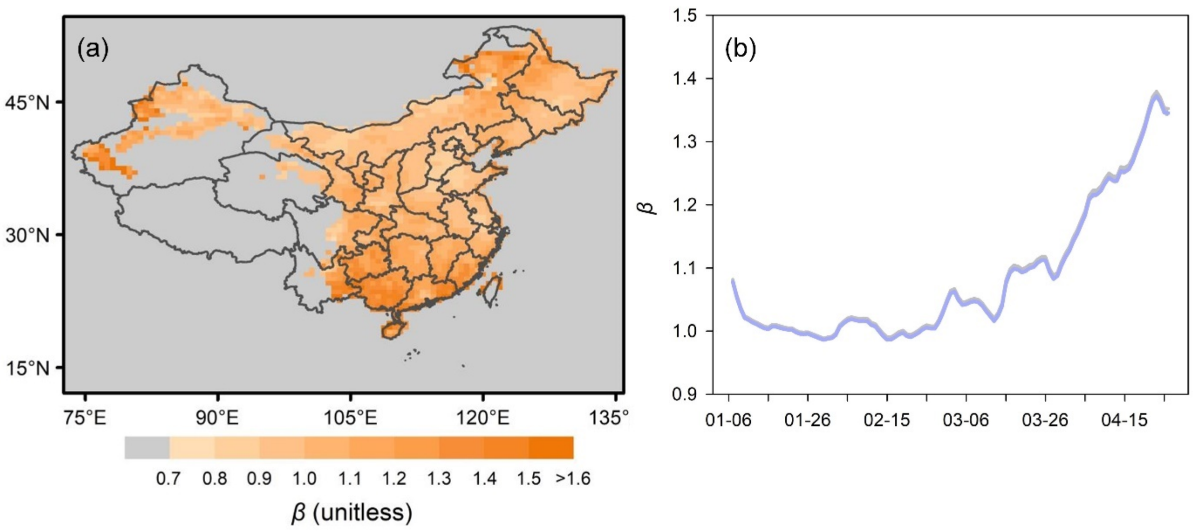

Fig. S7. (a) Spatial distribution and (b) daily variations of beta values over China estimated from GEOS-Chem model. The daily variations are also 10-day moving average.

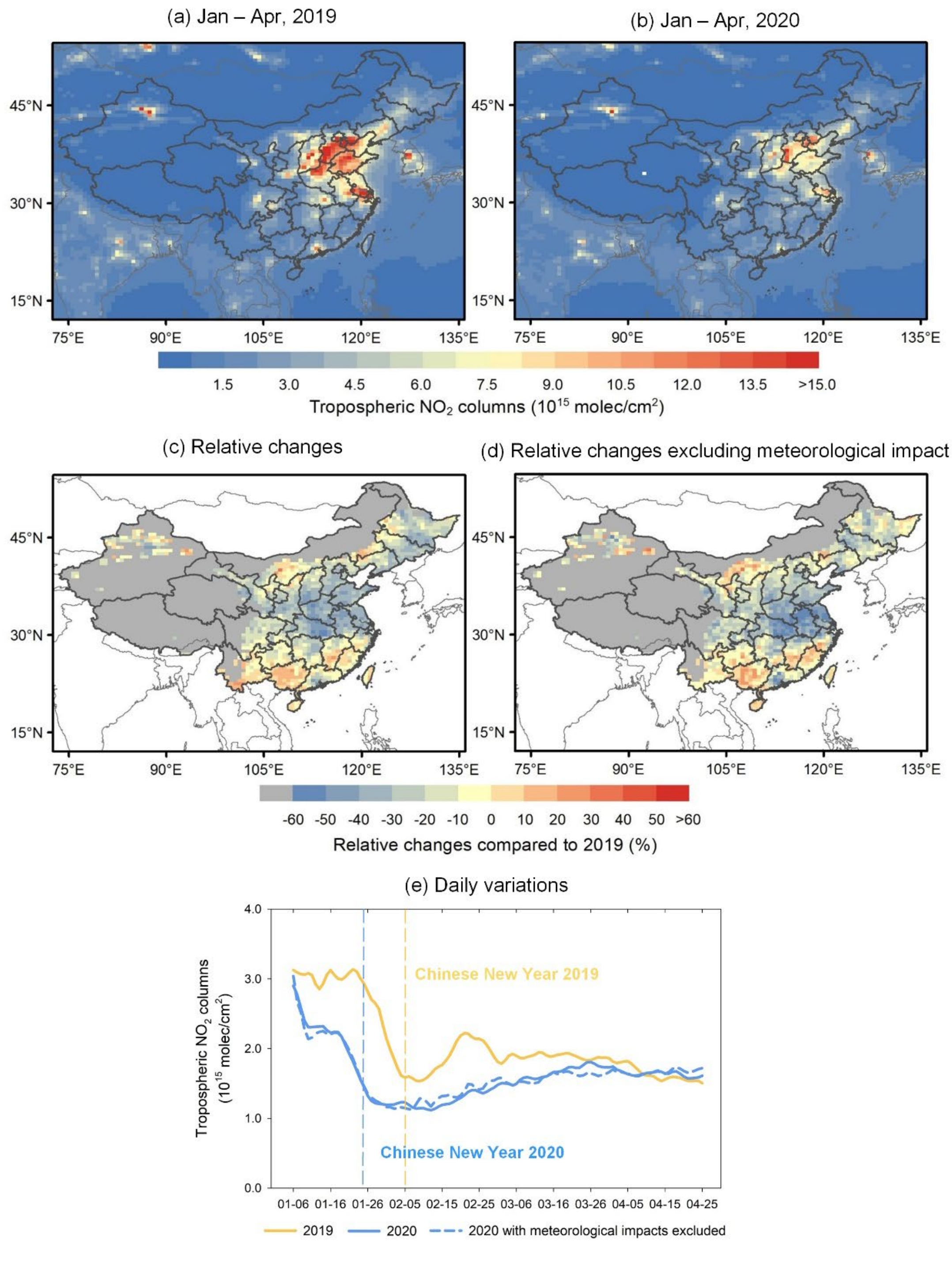

Fig. S8. Tropospheric NO_2 column densities from TROPOMI over China in 2019 and 2020. Average NO_2 TVCDs from Jan to Apr in (a) 2019 and (b) 2020. (c) Total relative differences between 2019 and 2020 and (d) Relative change between 2019 and 2020 excluding the influence from meteorological variations. (e) Daily variations in the 10-day moving average of NO_2 TVCDs from TROPOMI over China.

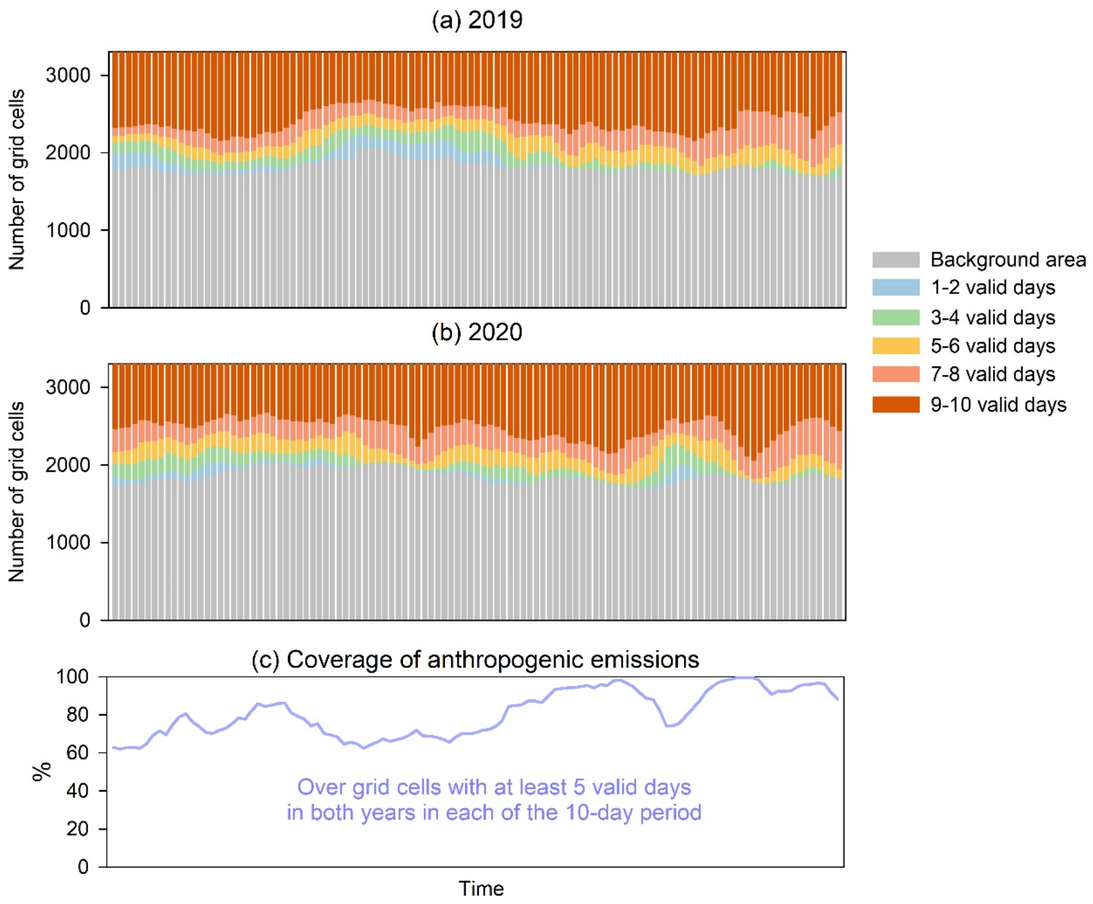

Fig. S9. Sample size in the 10-day moving average calculation of satellite NO_2 TVCDs and representation of national NO_x emissions. Number of grid cells that are filtered out (i.e., $<1\times10^{15}$ molec/cm^2) (grey) or have a certain number of valid days in (a) 2019 and (b) 2020. (c) The coverage of anthropogenic emissions on each day over grid cells with at least 5 valid days in both years in the 10-day moving average calculation.

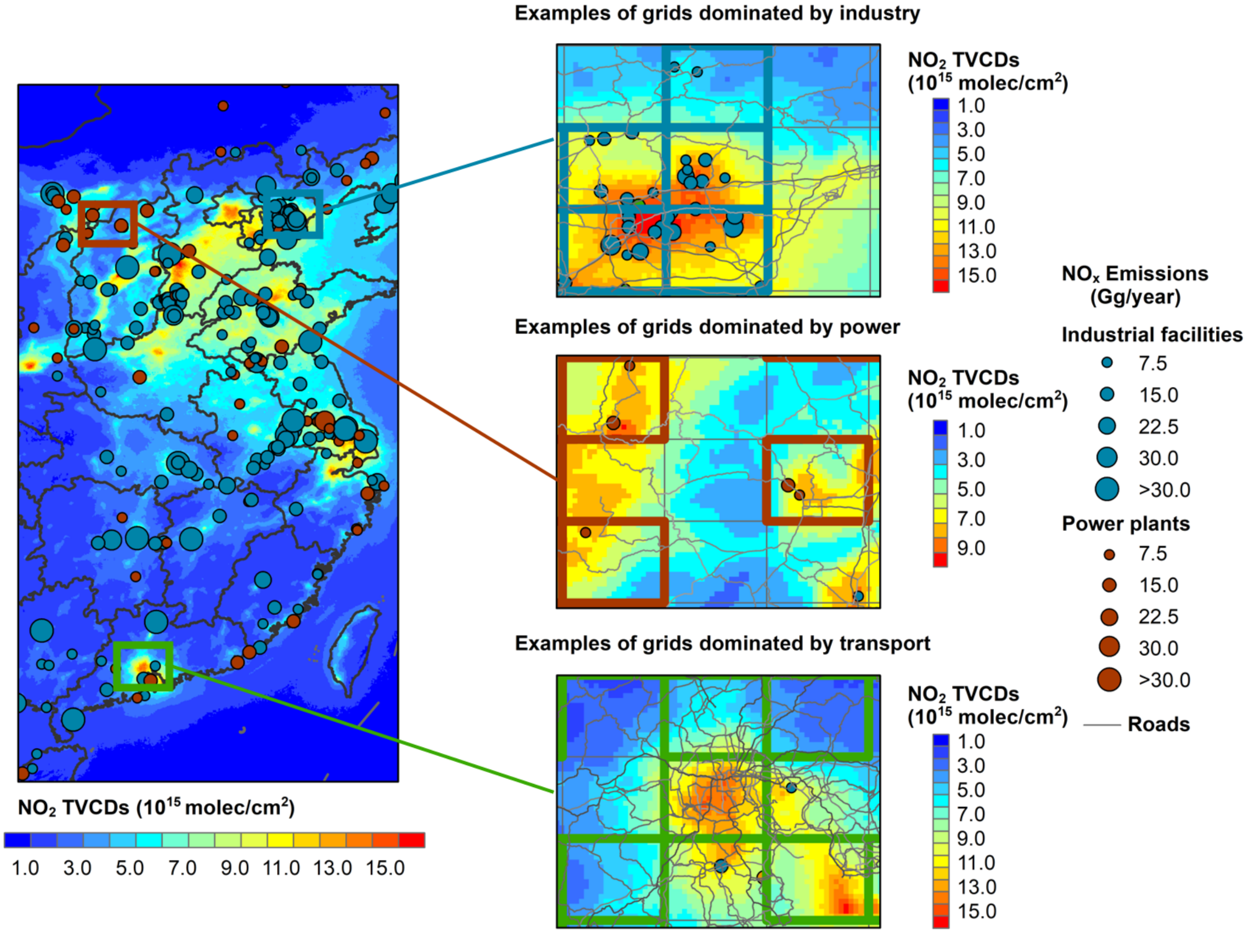

Fig. S10. TROPOMI NO_2 columns over the GEOS-Chem grid cells dominated by industry, power, and transport sectors. The dots represent the point sources of NO_x emissions from industrial facilities and power plants. We also show road networks in the figures.

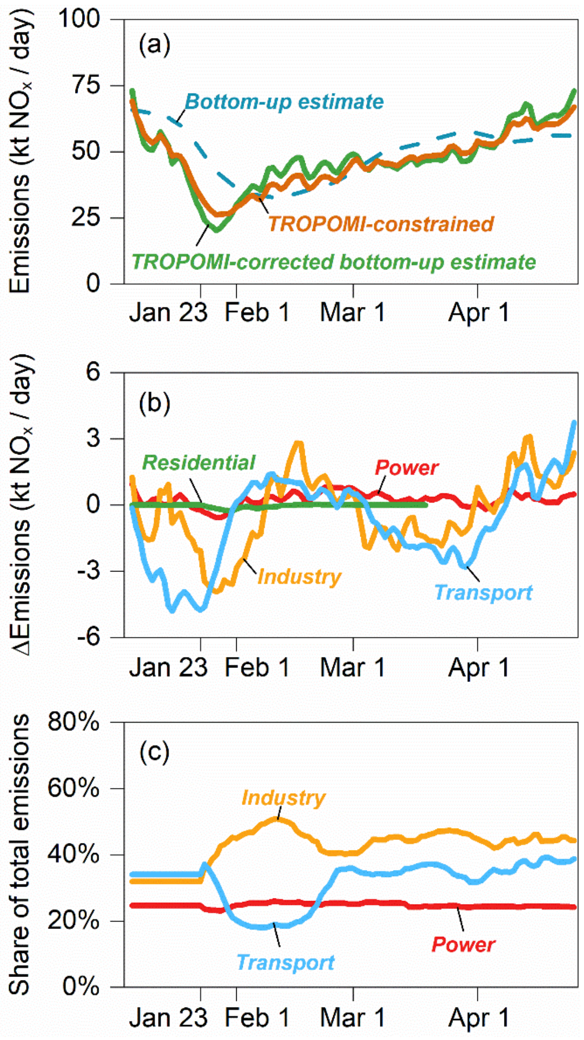

Fig. S11. Comparison of bottom-up NO_x emissions with the TROPOMI inversions in 2020. (a) Comparison of bottom-up estimated NO_x emissions, TROPOMI-constrained NO_x emissions, and TROPOMI-corrected bottom-up estimate. (b) The difference in NO_x emissions between the TROPOMI-constrained estimates and the bottom-up estimates over the grid cells dominated by each source sector. (c) The share of emissions from the grid cells dominated by one source sector in the total emissions of this sector. All of the data are shown in the 10-day moving average.

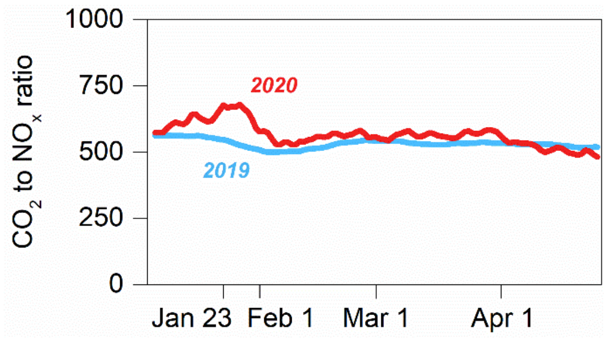

Fig. S12. Comparison of 10-day mean CO_2 to NO_x emission ratio between 2019 and 2020. The unit is g CO_2/g NO_2.

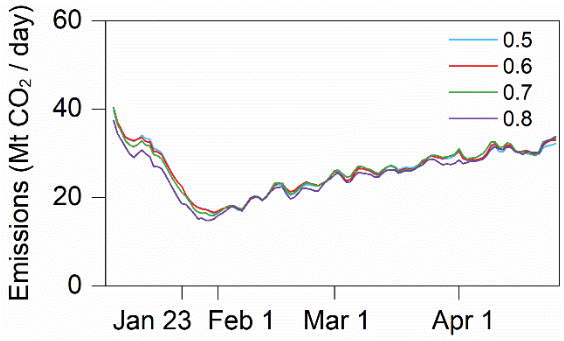

Fig. S13. The comparison of CO_2 inversion emissions derived from different threshold values for the definition of the dominant emission source sector.

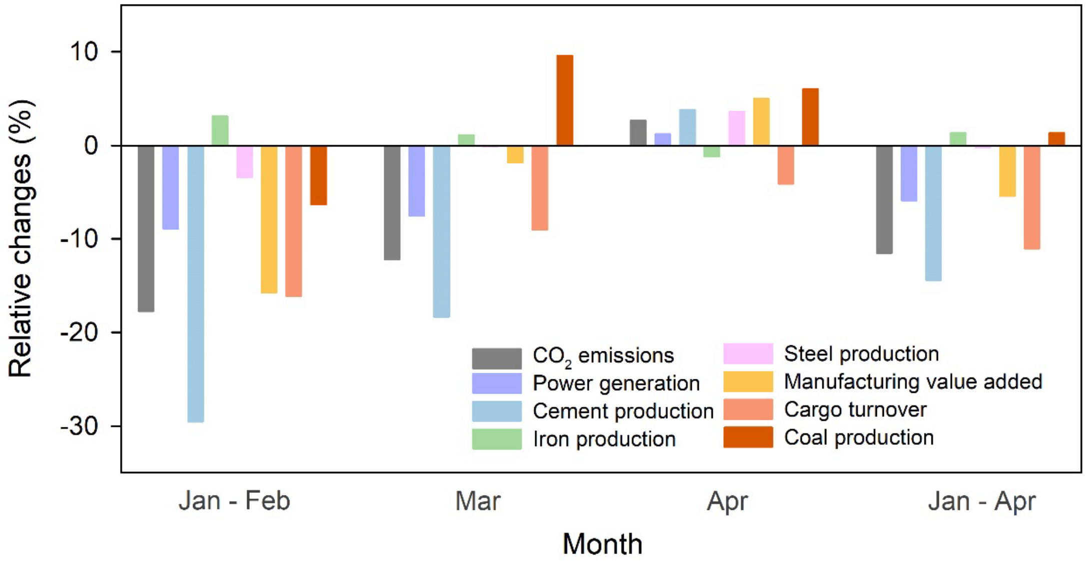

Fig. S14. Relative changes in CO_2 emissions and industrial activities from 2019 to 2020. The CO_2 emissions are derived from the TROPOMI-constrained emissions in this study. The other data that represent the industrial activities in China are all derived from the National Bureau of Statistics (http://www.stats.gov.cn/). The comparison between emissions and industrial indicators is made on monthly time scales for Jan-Feb, Mar, Apr, and Jan-Apr.

Table S1. Daily average emissions of NO_x and CO_2 for each month in 2019 and 2020

	NO_x (Mt NO_2)			CO_2 (Mt CO_2)		
**Month**	2019	2020	Diff	2019	2020	Diff
**January**	1.6	1.1	-26.9%	854.3	704.3	-17.6%
**February**	1.4	1.1	-23.3%	728.3	598.0	-17.9%
**March**	1.8	1.5	-17.0%	948.8	833.6	-12.1%
**April**	1.4	1.5	5.1%	736.2	755.8	2.7%
**January to April**	6.1	5.2	-15.9%	3267.6	2891.6	-11.5%

REFERENCES AND NOTES

1. H. Tian, Y. Liu, Y. Li, C.-H. Wu, B. Chen, M. U. G. Kraemer, B. Li, J. Cai, B. Xu, Q. Yang, B. Wang, P. Yang, Y. Cui, Y. Song, P. Zheng, Q. Wang, O. N. Bjornstad, R. Yang, B. T. Grenfell, O. G. Pybus, C. Dye, An investigation of transmission control measures during the first 50 days of the COVID-19 epidemic in China. *Science* **368**, 638–642 (2020).

2. IEA, *Global Energy Review 2020* (IEA, 2020); https://www.iea.org/reports/global-energy-review-2020.

3. F. Liu, A. Page, S. A. Strode, Y. Yoshida, S. Choi, B. Zheng, L. N. Lamsal, C. Li, N.A. Krotkov, H. Eskes, R. van der A, P. Veefkind, P. F. Levelt, O. P. Hauser, J. Joiner, Abrupt decline in tropospheric nitrogen dioxide over China after the outbreak of COVID-19. *Sci. Adv.* **6**, eabc2992 (2020).

4. Z. S. Venter, K. Aunan, S. Chowdhury, J. Lelieveld, COVID-19 lockdowns cause global air pollution declines with implications for public health risk. medRxiv 2020.04.10.20060673 (2020).

5. M. Bauwens, S. Compernolle, T. Stavrakou, J.-F. Müller, J. van Gent, H. Eskes, P. F. Levelt, R. van der A, J. P. Veefkind, J. Vlietinck, H. Yu, C. Zehner, Impact of coronavirus outbreak on NO_2 pollution assessed using TROPOMI and OMI observations. *Geophys. Res. Lett.* **47**, e2020GL087978 (2020).

6. C. Le Quéré, R. B. Jackson, M. W. Jones, A. J. P. Smith, S. Abernethy, R. M. Andrew, A. J. De-Gol, D. R. Willis, Y. Shan, J. G. Canadell, P. Friedlingstein, F. Creutzig, G. P. Peters, Temporary reduction in daily gAlobal CO_2 emissions during the COVID-19 forced confinement. *Nat. Clim. Chang.* **10**, 647–653 (2020).

7. G. Janssens-Maenhout, M. Crippa, D. Guizzardi, M. Muntean, E. Schaaf, F. Dentener, P. Bergamaschi, V. Pagliari, J. G. J. Olivier, J. A. H. W. Peters, J. A. van Aardenne, S. Monni, U. Doering, A. M. R. Petrescu, E. Solazzo, G. D. Oreggioni, EDGAR v4.3.2 Global Atlas of the three major greenhouse gas emissions for the period 1970–2012. *Earth Syst. Sci. Data* **11**, 959–1002 (2019).

8. G. P. Peters, G. Marland, C. Le Quéré, T. Boden, J. G. Canadell, M. R. Raupach, Rapid growth in CO_2 emissions after the 2008–2009 global financial crisis. *Nat. Clim. Chang.* **2**, 2–4 (2012).

9. M. McKee, D. Stuckler, If the world fails to protect the economy, COVID-19 will damage health not just now but also in the future. *Nat. Med.* **26**, 640–642 (2020).

10. D. Guan, D. Wang, S. Hallegatte, S. J. Davis, J. Huo, S. Li, Y. Bai, T. Lei, Q. Xue, D. Coffman, D. Cheng, P. Chen, X. Liang, B. Xu, X. Lu, S. Wang, K. Hubacek, P. Gong, Global supply-chain effects of COVID-19 control measures. *Nat. Hum. Behav.* **4**, 577–587 (2020).

11. P. Friedlingstein, M. W. Jones, M. O'Sullivan, R. M. Andrew, J. Hauck, G. P. Peters, W. Peters, J. Pongratz, S. Sitch, C. L. Quéré, D. C. E. Bakker, J. G. Canadell, P. Ciais, R. B. Jackson, P. Anthoni, L. Barbero, A. Bastos, V. Bastrikov, M. Becker, L. Bopp, E. Buitenhuis, N. Chandra, F. Chevallier, L. P. Chini, K. I. Currie, R. A. Feely, M. Gehlen, D. Gilfillan, T. Gkritzalis, D. S. Goll, N. Gruber, S. Gutekunst, I. Harris, V. Haverd, R. A. Houghton, G. Hurtt, T. Ilyina, A. K. Jain, E. Joetzjer, J. O. Kaplan, E. Kato, K. K. Goldewijk, J. I. Korsbakken, P. Landschützer, S. K. Lauvset, N. Lefèvre, A. Lenton, S. Lienert, D. Lombardozzi, G. Marland, P. C. McGuire, J. R. Melton, N. Metzl, D. R. Munro, J. E. M. S. Nabel, S.-I. Nakaoka, C. Neill, A. M. Omar, T. Ono, A. Peregon, D. Pierrot, B. Poulter, G. Rehder, L. Resplandy, E. Robertson, C. Rödenbeck, R. Séférian, J. Schwinger, N. Smith, P. P. Tans, H. Tian, B. Tilbrook, F. N. Tubiello, G. R. van der Werf, A. J. Wiltshire, S. Zaehle, Global carbon budget 2019. *Earth Syst. Sci. Data* **11**, 1783–1838 (2019).

12. J. Tollefson, How the coronavirus pandemic slashed carbon emissions—In five graphs. *Nature* **582**, 158–159 (2020).

13. Z. Liu, P. Ciais, Z. Deng, R. Lei, S. J. Davis, S. Feng, B. Zheng, D. Cui, X. Dou, P. He, B. Zhu, C. Lu, P. Ke, T. Sun, Y. Wang, X. Yue, Y. Wang, Y. Lei, H. Zhou, Z. Cai, Y. Wu, R. Guo, T. Han, J. Xue, O. Boucher, E. Boucher, F. Chevallier, Y. Wei, H. Zhong, C. Kang, N. Zhang, B. Chen, F. Xi, F. Marie, Q. Zhang, D. Guan, P. Gong, D. M. Kammen, K. He, H. J. Schellnhuber, COVID-19 causes record decline in global CO_2 emissions. arXiv:2004.13614 [econ.GN] (28 April 2020).

14. A. Eldering, P. O. Wennberg, D. Crisp, D. S. Schimel, M. R. Gunson, A. Chatterjee, J. Liu, F. M. Schwandner, Y. Sun, C. W. O'Dell, C. Frankenberg, T. Taylor, B. Fisher, G. B. Osterman, D.

Wunch, J. Hakkarainen, J. Tamminen, B. Weir, The Orbiting Carbon Observatory-2 early science investigations of regional carbon dioxide fluxes. *Science* **358**, eaam5745 (2017).

15. F. M. Schwandner, M. R. Gunson, C. E. Miller, S. A. Carn, A. Eldering, T. Krings, K. R. Verhulst, D. S. Schimel, H. M. Nguyen, D. Crisp, C. W. O'Dell, G. B. Osterman, L. T. Iraci, J. R. Podolske, Spaceborne detection of localized carbon dioxide sources. *Science* **358**, eaam5782 (2017).

16. S. Basu, S. Guerlet, A. Butz, S. Houweling, O. Hasekamp, I. Aben, P. Krummel, P. Steele, R. Langenfelds, M. Torn, S. Biraud, B. Stephens, A. Andrews, D. Worthy, Global CO_2 fluxes estimated from GOSAT retrievals of total column CO_2. *Atmos. Chem. Phys.* **13**, 8695–8717 (2013).

17. R. Nassar, T. G. Hill, C. A. McLinden, D. Wunch, D. B. A. Jones, D. Crisp, Quantifying CO_2 emissions from individual power plants from space. *Geophys. Res. Lett.* **44**, 10045–10053 (2017).

18. B. Zheng, F. Chevallier, P. Ciais, G. Broquet, Y. Wang, J. Lian, Y. Zhao, Observing carbon dioxide emissions over China's cities and industrial areas with the Orbiting Carbon Observatory-2. *Atmos. Chem. Phys.* **20**, 8501–8510 (2020).

19. J. P. Veefkind, I. Aben, K. McMullan, H. Förster, J. de Vries, G. Otter, J. Claas, H. J. Eskes, J. F. de Haan, Q. Kleipool, M. van Weele, O. Hasekamp, R. Hoogeveen, J. Landgraf, R. Snel, P. Tol, P. Ingmann, R. Voors, B. Kruizinga, R. Vink, H. Visser, P. F. Levelt, TROPOMI on the ESA Sentinel-5 Precursor: A GMES mission for global observations of the atmospheric composition for climate, air quality and ozone layer applications. *Remote Sens. Environ.* **120**, 70–83 (2012).

20. R. V. Martin, D. J. Jacob, K. Chance, T. P. Kurosu, P. I. Palmer, M. J. Evans, Global inventory of nitrogen oxide emissions constrained by space-based observations of NO_2 columns. *J. Geophys. Res. Atmos.* **108**, 4537 (2003).

21. S. Beirle, K. F. Boersma, U. Platt, M. G. Lawrence, T. Wagner, Megacity emissions and lifetimes of nitrogen oxides probed from space. *Science* **333**, 1737–1739 (2011).

22. B. Mijling, R. J. van der A, Using daily satellite observations to estimate emissions of short-lived air pollutants on a mesoscopic scale. *J. Geophys. Res. Atmos.* **117**, D17302 (2012).

23. S. Beirle, C. Borger, S. Dörner, A. Li, Z. Hu, F. Liu, Y. Wang, T. Wagner, Pinpointing nitrogen oxide emissions from space. *Sci. Adv.* **5**, eaax9800 (2019).

24. E. V. Berezin, I. B. Konovalov, P. Ciais, A. Richter, S. Tao, G. Janssens-Maenhout, M. Beekmann, E.-D. Schulze, Multiannual changes of CO_2 emissions in China: Indirect estimates derived from satellite measurements of tropospheric NO_2 columns. *Atmos. Chem. Phys.* **13**, 9415–9438 (2013).

25. I. B. Konovalov, E. V. Berezin, P. Ciais, G. Broquet, R. V. Zhuravlev, G. Janssens-Maenhout, Estimation of fossil-fuel CO_2 emissions using satellite measurements of "proxy" species. *Atmos. Chem. Phys.* **16**, 13509–13540 (2016).

26. F. Liu, B. N. Duncan, N. A. Krotkov, L. N. Lamsal, S. Beirle, D. Griffin, C. A. McLinden, D. L. Goldberg, Z. Lu, A methodology to constrain carbon dioxide emissions from coal-fired power plants using satellite observations of co-emitted nitrogen dioxide. *Atmos. Chem. Phys.* **20**, 99–116 (2020).

27. M. Reuter, M. Buchwitz, A. Hilboll, A. Richter, O. Schneising, M. Hilker, J. Heymann, H. Bovensmann, J. P. Burrows, Decreasing emissions of NO_x relative to CO_2 in East Asia inferred from satellite observations. *Nat. Geosci.* **7**, 792–795 (2014).

28. B. Zheng, D. Tong, M. Li, F. Liu, C. Hong, G. Geng, H. Li, X. Li, L. Peng, J. Qi, L. Yan, Y. Zhang, H. Zhao, Y. Zheng, K. He, Q. Zhang, Trends in China's anthropogenic emissions since 2010 as the consequence of clean air actions. *Atmos. Chem. Phys.* **18**, 14095–14111 (2018).

29. L. N. Lamsal, R. V. Martin, A. Padmanabhan, A. van Donkelaar, Q. Zhang, C. E. Sioris, K. Chance, T. P. Kurosu, M. J. Newchurch, Application of satellite observations for timely updates to global anthropogenic NO_x emission inventories. *Geophys. Res. Lett.* **38**, L05810 (2011).

30. Y. X. Wang, M. B. McElroy, D. J. Jacob, R. M. Yantosca, A nested grid formulation for chemical transport over Asia: Applications to CO. *J. Geophys. Res. Atmos.* **109**, D22307 (2004).

31. P. M. Forster, H. I. Forster, M. J. Evans, M. J. Gidden, C. D. Jones, C. A. Keller, R. D. Lamboll, C. Le Quéré, J. Rogelj, D. Rosen, C.-F. Schleussner, T. B. Richardson, C. J. Smith, S. T. Turnock, Current and future global climate impacts resulting from COVID-19. *Nat. Clim. Chang.* **10**, 913–919 (2020).

32. Center for International Earth Science Information Network - CIESIN - Columbia University. 2018. Gridded Population of the World, Version 4 (GPWv4): Population Count Adjusted to Match 2015 Revision of UN WPP Country Totals, Revision 11. Palisades, NY: NASA Socioeconomic Data and Applications Center (SEDAC). https://doi.org/10.7927/H4PN93PB.

33. H. Hersbach, B. Bell, P. Berrisford, S. Hirahara, A. Horányi, J. Muñoz-Sabater, J. Nicolas, C. Peubey, R. Radu, D. Schepers, A. Simmons, C. Soci, S. Abdalla, X. Abellan, G. Balsamo, P. Bechtold, G. Biavati, J. Bidlot, M. Bonavita, G. De Chiara, P. Dahlgren, D. Dee, M. Diamantakis, R. Dragani, J. Flemming, R. Forbes, M. Fuentes, A. Geer, L. Haimberger, S. Healy, R. J. Hogan, E. Hólm, M. Janisková, S. Keeley, P. Laloyaux, P. Lopez, C. Lupu, G. Radnoti, P. de Rosnay, I. Rozum, F. Vamborg, S. Villaume, J.-N. Thépaut, The ERA5 global reanalysis. *Q. J. R. Meteorol. Soc.* **146**, 1999–2049 (2020).

34. M. Crippa, E. Solazzo, G. Huang, D. Guizzardi, E. Koffi, M. Muntean, C. Schieberle, R. Friedrich, G. Janssens-Maenhout, High resolution temporal profiles in the Emissions Database for Global Atmospheric Research. *Sci. Data* **7**, 121 (2020).

35. R. Gelaro, W. McCarty, M. J. Suárez, R. Todling, A. Molod, L. Takacs, C. Randles, A. Darmenov, M. G. Bosilovich, R. Reichle, K. Wargan, L. Coy, R. Cullather, C. Draper, S. Akella, V. Buchard, A. Conaty, A. da Silva, W. Gu, G.-K. Kim, R. Koster, R. Lucchesi, D. Merkova, J. E. Nielsen, G. Partyka, S. Pawson, W. Putman, M. Rienecker, S. D. Schubert, M. Sienkiewicz, B. Zhao, The modern-era retrospective analysis for research and applications, version 2 (MERRA-2). *J. Climate* **30**, 5419–5454 (2017).

36. J.-T. Lin, M. B. McElroy, Impacts of boundary layer mixing on pollutant vertical profiles in the lower troposphere: Implications to satellite remote sensing. *Atmos. Environ.* **44**, 1726–1739 (2010).

37. M. Li, Q. Zhang, J.-i. Kurokawa, J.-H. Woo, K. He, Z. Lu, T. Ohara, Y. Song, D. G. Streets, G. R. Carmichael, Y. Cheng, C. Hong, H. Huo, X. Jiang, S. Kang, F. Liu, H. Su, B. Zheng, MIX: A mosaic Asian anthropogenic emission inventory under the international collaboration framework of the MICS-Asia and HTAP. *Atmos. Chem. Phys.* **17**, 935–963 (2017).

38. L. T. Murray, D. J. Jacob, J. A. Logan, R. C. Hudman, W. J. Koshak, Optimized regional and interannual variability of lightning in a global chemical transport model constrained by LIS/OTD satellite data. *J. Geophys. Res. Atmos.* **117**, D20307 (2012).

39. R. C. Hudman, N. E. Moore, A. K. Mebust, R. V. Martin, A. R. Russell, L. C. Valin, R. C. Cohen, Steps towards a mechanistic model of global soil nitric oxide emissions: Implementation and space based-constraints. *Atmos. Chem. Phys.* **12**, 7779–7795 (2012).

40. M. Liu, J. Lin, H. Kong, K. F. Boersma, H. Eskes, Y. Kanaya, Q. He, X. Tian, K. Qin, P. Xie, R. Spurr, R. Ni, Y. Yan, H. Weng, J. Wang, A new TROPOMI product for tropospheric NO_2 columns over East Asia with explicit aerosol corrections. *Atmos. Meas. Tech.* **13**, 4247–4259 (2020).

41. V. Shah, D. J. Jacob, K. Li, R. F. Silvern, S. Zhai, M. Liu, J. Lin, Q. Zhang, Effect of changing NO_x lifetime on the seasonality and long-term trends of satellite-observed tropospheric NO_2 columns over China. *Atmos. Chem. Phys.* **20**, 1483–1495 (2020).

42. M. Li, H. Liu, G. Geng, C. Hong, F. Liu, Y. Song, D. Tong, B. Zheng, H. Cui, H. Man, Q. Zhang, K. He, Anthropogenic emission inventories in China: A review. *Natl. Sci. Rev.* **4**, 834–866 (2017).

43. F. Liu, Q. Zhang, D. Tong, B. Zheng, M. Li, H. Huo, K. B. He, High-resolution inventory of technologies, activities, and emissions of coal-fired power plants in China from 1990 to 2010. *Atmos. Chem. Phys.* **15**, 13299–13317 (2015).

44. D. Tong, Q. Zhang, F. Liu, G. Geng, Y. Zheng, T. Xue, C. Hong, R. Wu, Y. Qin, H. Zhao, L. Yan, K. He, Current emissions and future mitigation pathways of coal-fired power plants in China from 2010 to 2030. *Environ. Sci. Technol.* **52**, 12905–12914 (2018).

45. B. Zheng, Q. Zhang, S. J. Davis, P. Ciais, C. Hong, M. Li, F. Liu, D. Tong, H. Li, K. He, Infrastructure shapes differences in the carbon intensities of Chinese cities. *Environ. Sci. Technol.* **52**, 6032–6041 (2018).

46. B. Zheng, H. Huo, Q. Zhang, Z. L. Yao, X. T. Wang, X. F. Yang, H. Liu, K. B. He, High-resolution mapping of vehicle emissions in China in 2008. *Atmos. Chem. Phys.* **14**, 9787–9805 (2014).

47. L. Peng, Q. Zhang, Z. Yao, D. L. Mauzerall, S. Kang, Z. Du, Y. Zheng, T. Xue, K. He, Underreported coal in statistics: A survey-based solid fuel consumption and emission inventory for the rural residential sector in China. *Appl. Energy* **235**, 1169–1182 (2019).

48. B. Zheng, Q. Zhang, D. Tong, C. Chen, C. Hong, M. Li, G. Geng, Y. Lei, H. Huo, K. He, Resolution dependence of uncertainties in gridded emission inventories: A case study in Hebei, China. *Atmos. Chem. Phys.* **17**, 921–933 (2017).

49. G. Geng, Q. Zhang, R. V. Martin, J. Lin, H. Huo, B. Zheng, S. Wang, K. He, Impact of spatial proxies on the representation of bottom-up emission inventories: A satellite-based analysis. *Atmos. Chem. Phys.* **17**, 4131–4145 (2017).

50. M. Li, Z. Klimont, Q. Zhang, R. V. Martin, B. Zheng, C. Heyes, J. Cofala, Y. Zhang, K. He, Comparison and evaluation of anthropogenic emissions of SO_2 and NO_x over China. *Atmos. Chem. Phys.* **18**, 3433–3456 (2018).
